# Engineering of a genome-reduced strain *Bacillus amyloliquefaciens* for enhancing surfactin production

**DOI:** 10.1186/s12934-020-01485-z

**Published:** 2020-12-07

**Authors:** Fang Zhang, Kaiyue Huo, Xingyi Song, Yufen Quan, Shufang Wang, Zhiling Zhang, Weixia Gao, Chao Yang

**Affiliations:** 1 Key Laboratory of Molecular Microbiology and Technology for Ministry of Education, Key Laboratory of Bioactive Materials for Ministry of Education, College of Life Sciences, Nankai University, Tianjin, China; 2 Department of Oral and Maxillofacial Radiology, Tianjin Stomatological Hospital, School of Medicine, Nankai University, Tianjin, 300041 China; 3 Tianjin Key Laboratory of Oral and Maxillofacial Function Reconstruction, Tianjin, 300041 China; 4 MOE Key Laboratory of Industrial Fermentation Microbiology, College of Biotechnology, Tianjin University of Science and Technology, Tianjin, China

**Keywords:** *Bacillus amyloliquefaciens*, Genome reduction, Promoter engineering, Surfactin production

## Abstract

**Background:**

Genome reduction and metabolic engineering have emerged as intensive research hotspots for constructing the promising functional chassis and various microbial cell factories. Surfactin, a lipopeptide-type biosurfactant with broad spectrum antibiotic activity, has wide application prospects in anticancer therapy, biocontrol and bioremediation. *Bacillus amyloliquefaciens* LL3, previously isolated by our lab, contains an intact *srfA* operon in the genome for surfactin biosynthesis.

**Results:**

In this study, a genome-reduced strain GR167 lacking ~ 4.18% of the *B. amyloliquefaciens* LL3 genome was constructed by deleting some unnecessary genomic regions. Compared with the strain NK-1 (LL3 derivative, Δ*upp*ΔpMC1), GR167 exhibited faster growth rate, higher transformation efficiency, increased intracellular reducing power level and higher heterologous protein expression capacity. Furthermore, the chassis strain GR167 was engineered for enhanced surfactin production. Firstly, the iturin and fengycin biosynthetic gene clusters were deleted from GR167 to generate GR167ID. Subsequently, two promoters PR_*suc*_ and PR_*tpxi*_ from LL3 were obtained by RNA-seq and promoter strength characterization, and then they were individually substituted for the native *srfA* promoter in GR167ID to generate GR167IDS and GR167IDT. The best mutant GR167IDS showed a 678-fold improvement in the transcriptional level of the *srfA* operon relative to GR167ID, and it produced 311.35 mg/L surfactin, with a 10.4-fold increase relative to GR167.

**Conclusions:**

The genome-reduced strain GR167 was advantageous over the parental strain in several industrially relevant physiological traits assessed and it was highlighted as a chassis strain for further genetic modification. In future studies, further reduction of the LL3 genome can be expected to create high-performance chassis for synthetic biology applications.

## Background

With the development of systems and synthetic biology, numerous studies have focused on the design and construction of the optimal functional microbial chassis with reduced genomes and superior physiological characteristics [1, 2]. Moderate genome reduction can create synthetic biology chassis with optimized genomic sequences, efficient metabolic regulatory networks and superior cellular physiological characteristics [3–5]. So far, several model microorganisms, such as *Escherichia coli* [6], *Bacillus subtilis* [7, 8] and *Pseudomonas putida* [9], have been intensively researched for minimal genome construction due to their clear genetic background and efficient genome editing approaches.

Surfactin, which contains a ring-shaped heptapeptide and a β-hydroxy fatty acid chain with 13–16 carbons, is a cyclic lipopeptide (CLP) biosurfactant with broad-spectrum antibiotic activity and mainly produced by *Bacillus* sp. via multifunctional non-ribosome peptide synthases (NRPSs) encoded by the *srfA* operon containing four open reading frames (*srfAA*, *srfAB*, *srfAC* and *srfAD*) [10, 11]. Surfactin is one of the most promising green biosurfactants due to its anti-viral, anti-tumor and anti-bacterial activities, which can be used in various fields, such as food processing, pharmaceuticals, oil recovery, and environmental governance [12–14].

In recent years, several metabolic engineering strategies have been proposed for enhancing biosurfactant production, mainly including promoter engineering [15–17], the reduction of by-product formation [11], the enhancement of the precursor supply [2], the improvement of biosurfactant transmembrane efflux [18], and the modification of global regulatory factors [19]. Among which, promoter engineering is highlighted as a powerful tool for enhancing the titer of biosurfactants. For example, the titer of iturin A was increased from an undetectable level to 37.35 mg/L by inserting a strong C2up promoter upstream of the *itu* operon in *B. amyloliquefaciens*. [17] In another study, the titer of surfactin in *B. subtilis* was elevated from 0.07 g/L to 0.26 g/L by the replacement of the native *srfA* promoter with a constitutive promoter P_*veg*_ [20]. In addition to the natural promoters, Jiao et al. [16] developed a chimeric promoter Pg3 for driving the synthesis of surfactin, resulting in a 15.6-fold increase in the titer of surfactin relative to the wild-type *B. subtilis* THY-7. However, efficient promoters need to be explored for the enhancement of biosurfactants production by members of the genus *Bacillus*.

Currently, endogenous promoters are highlighted as promising candidates for improved production of bacterial secondary metabolites [21]. For example, 14 endogenous promoters identified from *Streptomyces albus* J1074 by RNA-seq and reporter assays were successfully used to activate a cryptic gene cluster in *S. griseu* [22]. In another study, four endogenous promoters identified from *S. coelicolor* M145 by RNA-seq and reporter assays were used to activate cryptic biosynthetic clusters for jadomycin B production in *S. venezuelae* ISP5230 [5].

*B. amyloliquefaciens* LL3 was isolated initially for poly-γ-glutamic acid (γ-PGA) production by our lab, and whole genome of LL3 is currently available in the GenBank database (accession no. NC_017190.1) [23]. LL3 has a genomic size of 3,995,227 bp with an average G+C content of 45.7% and a circular plasmid (pMC1) of 6758 bp. In particular, an intact *srfA* operon was found in the genome of LL3, suggesting the capability for surfactin biosynthesis. The essential genes and genomic islands (GIs) in LL3 were also identified by the Essential Genes Database (http://tubic.tju.edu.cn/deg/) and GIs Analysis Software (http://tubic.tju.edu.cn/GC-Profile/). Previously, a marker-free large fragments deletion method was well established in LL3 [24]. Therefore, previous studies have laid a foundation for genome reduction and enhanced surfactin production in LL3.

**Fig. 1:**
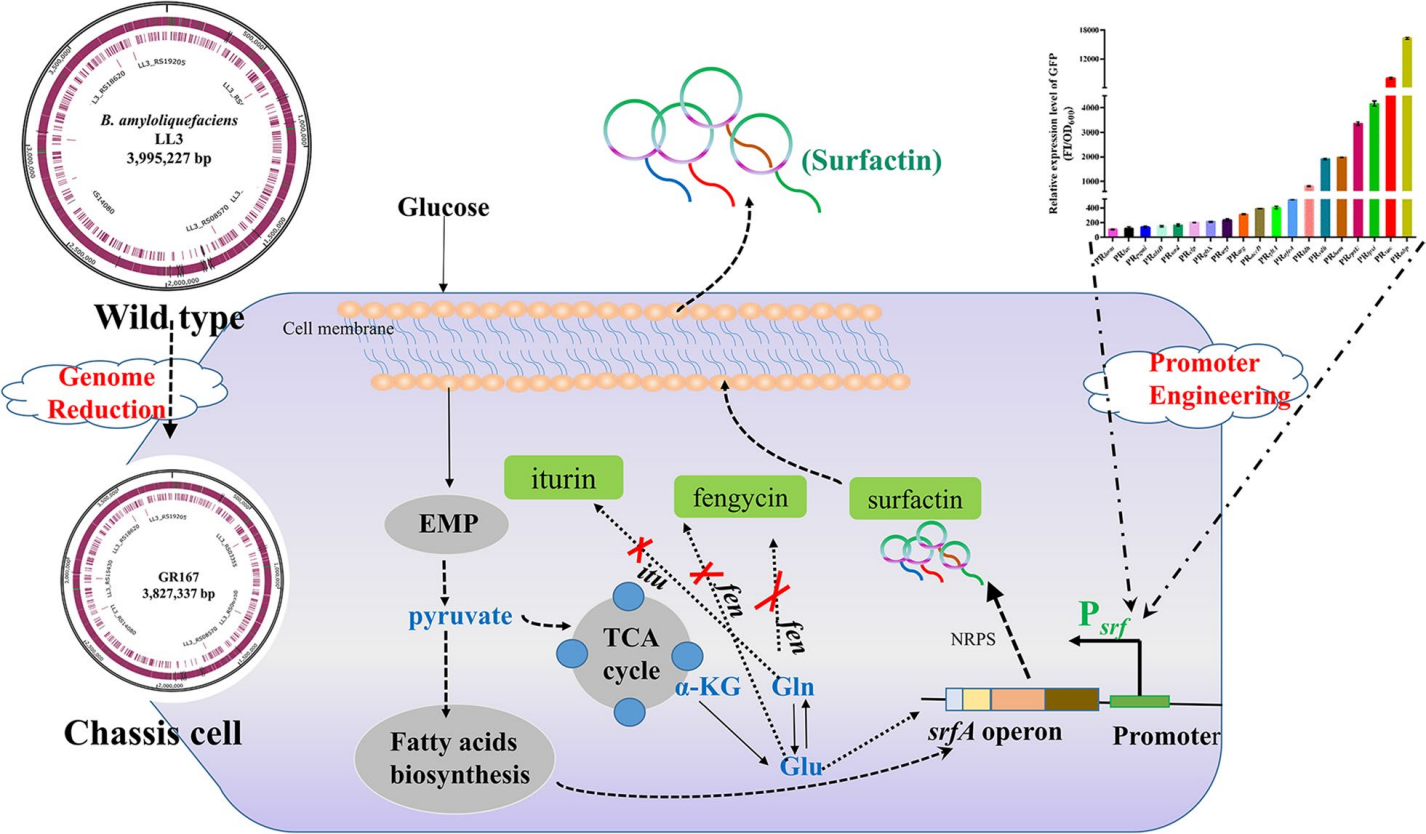
Schematic diagram of the overall strategy for enhancing lipopeptide surfactin yield in *B. amyloliquefaciens*

In this study, a genome-reduced strain GR167 was constructed from *B. amyloliquefaciens* NK-1 (LL3 derivative, Δ*upp*ΔpMC1) [25] and evaluated as a functional chassis strain for several physiological traits. Furthermore, GR167 was engineered using metabolic engineering strategies for enhanced surfactin production. Strategies designed for enhancing surfactin production in *B. amyloliquefaciens* are shown in Fig. 1.

## Results and discussion

### Construction of genome-reduced *B. amyloliquefaciens* strains

**Fig. 2:**
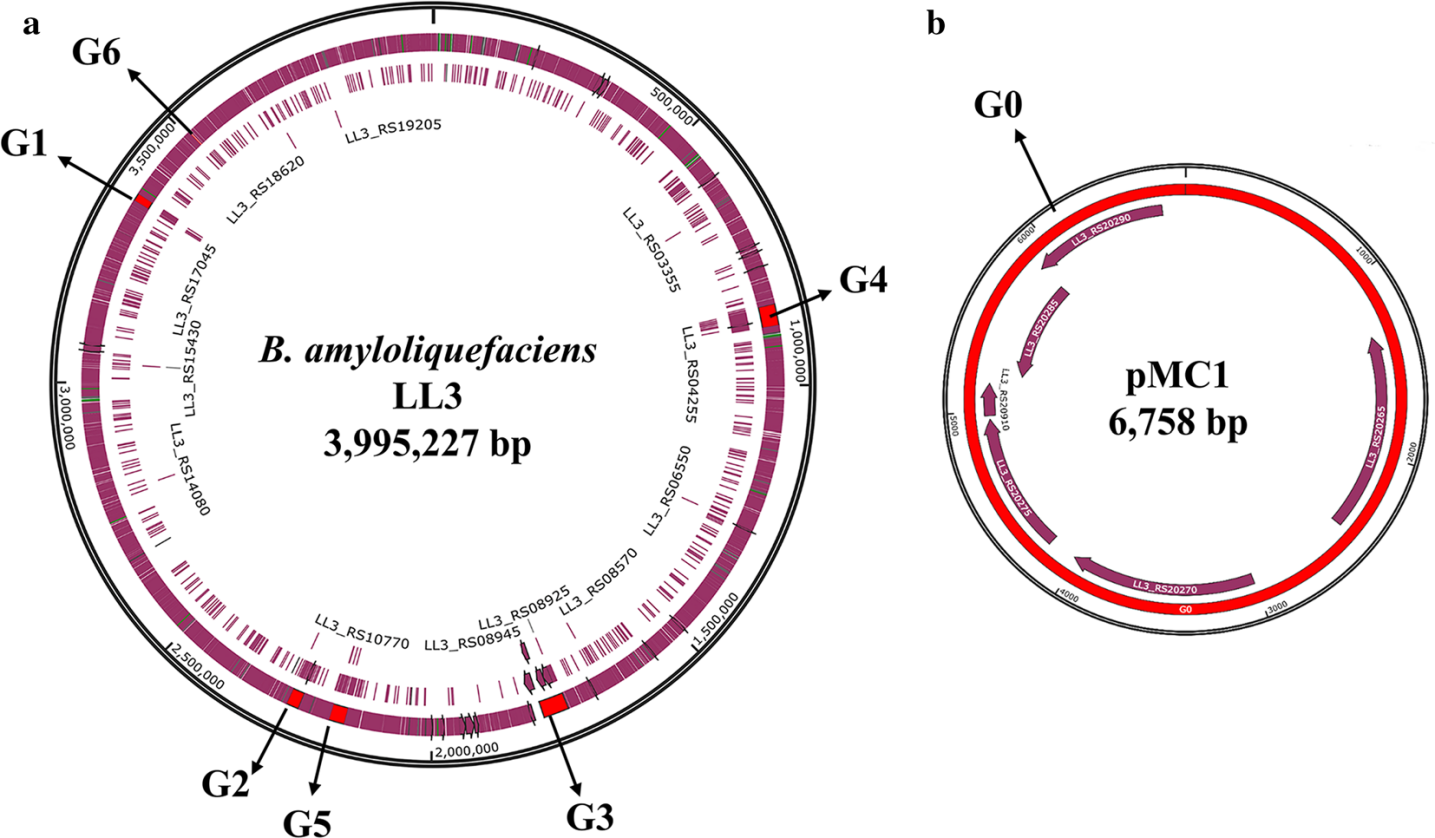
The construction of genome-reduced strain GR167. **a** The exact coordinates of the deleted regions (G1 to G6) on the chromosome of *B. amyloliquefaciens* LL3;** b** the physical map of the cured endogenous plasmid pMC1

To adapt to the adverse environmental conditions, there is a common mechanism horizontal gene transfer (HGT) among microorganisms, enabling host bacteria to acquire larger DNA segments, i.e., GIs, the G+C contents of which are significantly different from that of the core genome [26]. GIs usually carry some functional genes related to pathogenicity and antibiotic resistance, leading to the emergence of multiple resistant bacteria by HGT [27]. In addition, there are latent secondary metabolic biosynthesis gene clusters scattered across the LL3 genome, which may increase the metabolic burden on cells and the purification cost of target products [28]. Consequently, to streamline the genome of LL3, the GIs containing putative protein genes, antibiotic biosynthesis genes and prophage protein genes, where the G+C contents deviate significantly from 45.7%, were selected as the knockout targets. Besides, the gene clusters *eps*, *bae* and *pgsBCA* responsible for the biosynthesis of extracellular polysaccharides, bacillaene and γ-PGA, respectively, which consume more energy and substrates, were also deleted from the LL3 genome. The detailed information on the deleted regions is summarized in Tables S1 and S2. The schematic diagram for deletion of large genomic segments in LL3 is presented in Additional file 1: Figure S1. Overall, a genome-reduced strain GR167 lacking ~ 4.18% of the LL3 genome was generated from NK-1 via a markerless deletion method [24]. The exact coordinates (G1 to G6) of the deleted regions on chromosome and the physical map of the endogenous plasmid pMC1 are shown in Fig. 2a, b, respectively.

Deleting redundant genes from a bacterial genome is expected to create superior chassis cells for the industrial production of valuable bio-based chemicals. Due to the existence of unannotated genes in the LL3 genome and lack of insight into the interactions among known genes, several industrially-relevant physiological traits were evaluated in GR167 to determine whether a chassis strain with excellent characteristics can be produced by genome reduction.

### Genome reduction can improve the growth rate of LL3

**Fig. 3:**
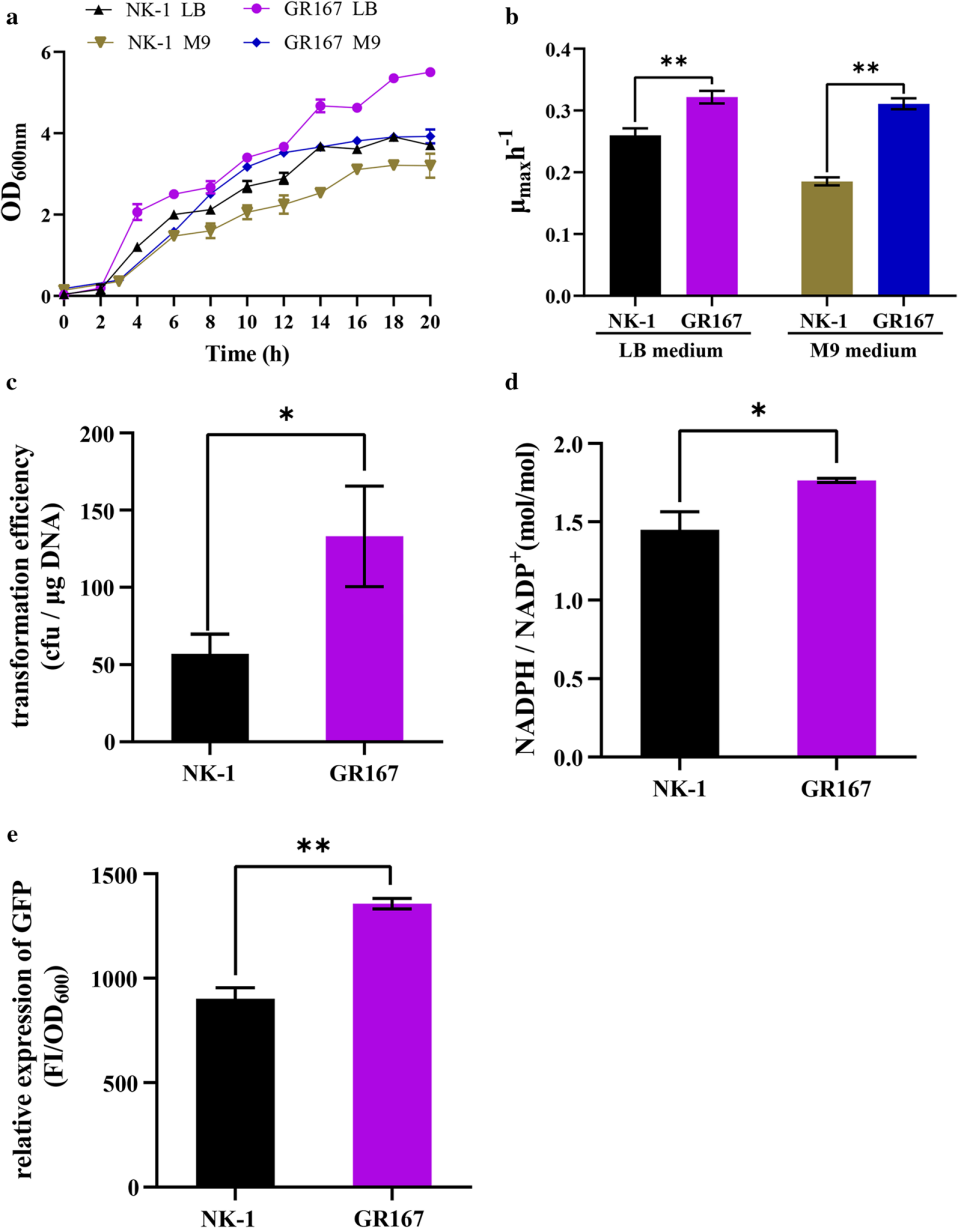
Physiological characteristics assessment of strains. **a** Growth curves measured in LB medium; **b** growth curves measured in M9 medium;** c** the maximum specific growth rate (*μ*_max_) measured in LB medium; **d** the *μ*_max_ measured in M9 medium; **e** transformation efficiency; **f** intracellular reducing power level (NADPH/NADP^+^ molar ratio); **g** The relative fluorescence intensities (GFP, green fluorescent protein; FI, fluorescence intensity). Values denote mean ± SD of triplicates (*P < 0.05, **P < 0.01)

To evaluate the effect of non-essential genomic sequences on cell growth, the growth profiles of GR167 and the parental NK-1 strain were detected by following the optical density (OD_600_) of cells cultured in both poor (M9 medium) and rich (LB medium) conditions. As shown in Fig. 3a, obviously, whether incubated in LB or M9 medium, GR167 grew faster and yielded higher biomass with approximately 1.5- and 1.2-fold higher at the plateau phase than that of NK-1, respectively. The maximum specific growth rate (*μ*_max_) of the strains was further determined during exponential growth. When compared with NK-1, the GR167 showed a 23.7% and 67% increase in *μ*_max_ when cultured in LB and M9 medium, respectively (Fig. 3b).

During the evolution of bacteria various processes (e.g., horizontal gene transfer, HGT) enlarge the genome size, which may be unfavorable to cell growth because of the extra consumption for the expression of redundant metabolic pathways [29]. In current study, there was a positive correlation between cell growth and cumulative deletions, and deleting ~ 4.18% of the LL3 genome did not affect cellular viability of GR167. Moreover, the growth rate of GR167 outcompeted the parental strain under the tested culture conditions, making it a candidate chassis cell for further genetic engineering.

### Genome reduction can enhance transformation efficiency

An ideal chassis cell is expected to possess the excellent capacity to take up exogenous plasmids. As shown in Fig. 3c, GR167 surpassed the transformation efficiency of the parental strain NK-1 by about 133%, indicating that the GR167 presented a better DNA uptake state during electroporation. Since the transformation efficiency is a synergistic effect caused by many factors [30], it is difficult to pinpoint which particular removed genes affected the observed results.

### Genome reduction can increase intracellular reducing power and the heterologous protein expression capacity

The intracellular reducing power (NADPH/NADP^+^), which is indispensable for basic anabolic processes [31], was measured in this study. The intracellular NADPH/NADP^+^ ratio of GR167 increased by 21.4% compared to the parental strain, (Fig. 3d), which may be attributed to the deletion of some NADPH-consuming biosynthesis pathways such as γ-PGA biosynthesis [32]. The improvement of intracellular reducing power level may be beneficial for GR167 to enhance the production of secondary metabolites.

Besides, an ideal chassis is expected to possess high heterologous protein expression capacity. In previous studies, green fluorescent protein (GFP) was selected as a model heterologous protein in genome-reduced *P. putida* KT2440 mutants, and the expression capacity of heterologous protein was characterized by the GFP fluorescence per biomass unit [9, 33]. In this study, the production capability of GFP was evaluated in GR167 by transcriptional level and the fluorescence intensity. As shown in Fig. 3e, when transformed with plasmid pHT-P_43_-*gfp*, the relative fluorescence intensity of GR167 was about 50.4% higher than that of NK-1 and the increase in the transcriptional level of *gfp* was also observed in GR167. Overall, genome reduction had a positive effect on improving the heterologous protein expression capacity of GR167.

### Genome reduction can improve the metabolic capacity

To further evaluate the changes of the metabolic phenotypes caused by genome reduction, a BIOLOG assay was used to test and analyze the overall utilization of the substrates by both NK-1 and GR167. In a total of 71 carbon sources, 23 carbon sources could be efficiently utilized by both strains. Compared to NK-1, GR167 showed a higher metabolic activity towards l-aspartic acid, citric acid, l-malic acid, l-glutamic acid and l-lactic acid (Table 1).

**Table 1:** Metabolic phenotype analysis of NK-1 and GR167

Substrate	NK-1	GR167
Methyl pyruvate	94	118
l-Aspartic acid	178	232
Tween 40	77	79
d-Lactic acid methyl ester	61	67
Citric acid	245	254
l-Malic acid	227	241
Formic acid	90	105
Acetic acid	82	87
d-Sorbitol	129	94
d-Maltose	138	106
d-Trehalose	198	154
d-Cellobiose	195	146
Gentiobiose	84	78
Sucrose	191	155
α-d-lactose	140	113
α-d-glucose	208	161
d-Mannose	185	146
d-Fructose	74	83
Glycerol	255	174
l-Glutamic acid	229	231
l-Lactic acid	191	193
γ-amino-butyric acid	144	113
Acetoacetic acid	112	108

The values denote mean of triplicates

### Use of genome-reduced strain GR167 as a starting strain for surfactin production

Surfactin is synthesized by a NRPS encoded by *srfA* operon (*srfAA*, *srfAB*, *srfAC* and *srfAD*) in microbes using four amino acids (l-glutamate, l-leucine, l-valine and l-aspartate) and fatty acids as precursors via a complex mechanism [34] (Additional file 1: Figure S4). For surfactin, it can hardly achieve a significant breakthrough in production only through traditional fermentation optimization because of its low yield in wild strains [16, 35]. Engineering and modifying microbial chassis could maximize its practical application ranges and obtain maximum theoretical yields of bio-based products of interests. Such as *B. subtilis* BSK814, a genome-reduced strain, was endowed with the ability to hyperproduce guanosine as well as acetoin by modifying different metabolic pathways [4, 19].

In this study, the chassis GR167 with the intact *srfA* operon and superior physiological traits was used as a starting strain for surfactin production. Because the fermentation broth of the NK-1 strain was too viscous to obtain a relatively purer surfactin product, the quantification of surfactin produced by NK-1 was very difficult. The γ-PGA production leads to the high viscosity and the limitation of dissolved oxygen of the culture broth [36] and competes with surfactin production for the common substrate glutamate (Glu) (Additional file 1: Figure S4). Moreover, both γ-PGA and surfactin are extracellular secretion products. Therefore, an extremely low yield of surfactin was detected with NK-1. Consequently, the mutant strain NK-ΔLP (NK-1 derivative, Δ*pgsBCA*) [37] without γ-PGA production was used as a control in the case of surfactin production. Surfactin produced by GR167 and NK-ΔLP was demonstrated by high-performance liquid chromatography-mass spectrometry (HPLC–MS). A slight increase (approximately 9.7%) in the surfactin titer was observed with GR167 (Additional file 1: Figure S2). Genome reduction seems to have little positive effects on the surfactin yield, however, the chassis GR167 constructed in this study shows superior genetic operability, e.g., higher transformation efficiency. In addition, higher growth rate of GR167 is also a critical factor for ensuring that further genetic modifications are successfully performed. Therefore, it is interesting and necessary to explore whether microbial cell factories with high surfactin production capabilities can be constructed by further modification of GR167.

### Characterization of surfactin produced by GR167

**Fig. 4:**
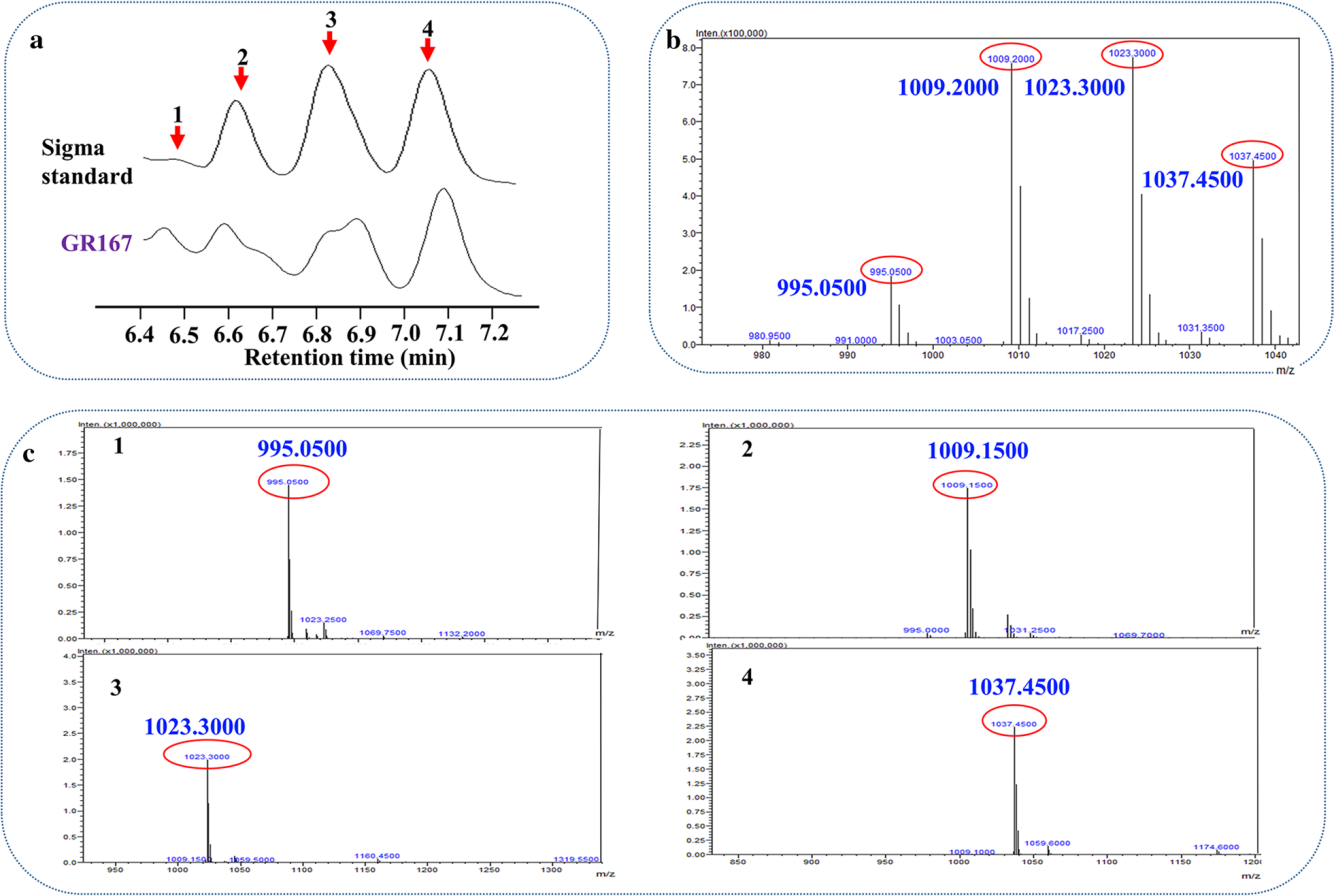
HPLC–MS chromatograms of surfactin standard and the samples produced by GR167. **a** HPLC chromatograms of surfactin from standard and GR167; **b** MS (mass spectra) of surfactin standard (Sigma); **c** MS of purified surfactin homologs produced by GR167. The peaks 1, 2, 3, and 4 products corresponding to the molecular ion peaks at m/z 995, 1009, 1023, and 1037, respectively

It was reported that surfactin produced by microorganisms is a mixture of several surfactin homologs [38]. In current study, by comparing the HPLC spectrogram of the produced surfactin by GR167 with that of the surfactin standard, there were four peaks to be detected within a retention time range of 6.4 to 7.2 min (Fig. 4a). To determine precisely the surfactin purity produced by GR167, each peak product of GR167 was purified from the culture supernatant and subjected to mass spectra (MS) analysis. The mass spectra of the product peaks 1, 2, 3, and 4 had the molecular ion peaks at *m/z* 995, 1009, 1023, and 1037, which were attributed to [C_13_ + 2H]^2+^, [C_14_ + 2H]^2+^, [C_15_ + 2H]^2+^, and [C_16_ + 2H]^2+^, respectively (Fig. 4b, c). These compounds are four homologs of surfactin, which differ in their β-hydroxy fatty acid chain length by a CH_2_ group of 14 Da.

### Enhancing surfactin production by blocking the potential competitive pathways

A transcriptional comparison between *B. amyloliquefaciens* LL3 and NK-ΔLP using RNA-seq revealed that the transcriptional levels of the gene clusters *srfA*, *itu* and *fen*, responsible for surfactin, iturin A and fengycin biosynthesis were all up-regulated when *pgsBCA* was removed (Additional file 1: Figure S3). Iturin A and fengycin belonging to CLP antibiotics are structural analogues of surfactin [39], possibly leading to the reduction of the purity of the extracted surfactin from the culture supernatant. Iturin A and fengycin are synthesized by NRPSs like surfactin [13]; thus, they may share similar biosynthesis mechanisms with surfactin and their biosynthesis may compete for NADPH, energy and direct precursors with surfactin biosynthesis. In this study, the gene clusters *itu* (37.2 kb) and *fen* (11.5 kb) were deleted to enhance surfactin production. The resulting mutants were designated as GR167I (Δ*itu*), GR167D (Δ*fen*) and GR167ID (Δ*itu*, Δ*fen*). The titer of surfactin was increased to 32.88 mg/L in GR167ID, with a 10% and 56% improvement in the titer and specific productivity of surfactin compared to GR167, respectively (Additional file 1: Figure S2). Analysis of surfactin synthesis pathway allows to assume that blocking the potential competitive pathways can eliminate the competition for the same amino acid precursors, allowing for the redistribution of substrates and precursors towards surfactin biosynthesis (Additional file 1: Figure S4).

### Construction of endogenous promoter library of *B. amyloliquefaciens* LL3

Promoter engineering is considered as a promising approach for enhanced production of bacterial secondary metabolites [21, 22]. FPKM (fragments per kilobase million) value is positively correlated with the transcriptional activity of a gene [40], which therefore can be regarded as an indicator for initial screening of promoters. Through RNA-seq analysis of LL3, all genes were ranked and classified into three groups based on their FPKM values, i.e., lower than 1250, 1250–4000 and higher than 4000. Then, the first six genes with higher FPKM values in each group were selected, and their upstream regions were predicted and cloned as described in “Methods”, named PR_*x*_ [*x*: the name of various related genes; PR: the sequences of predicted promoters with their ribosomal binding sites (RBSs)] and represented weak, moderate and strong promoters, respectively (Additional file 1: Table S3). Subsequently, various reporter gene vectors derived from pHT01 containing fused fragments of the predicted promoters and *gfp* gene were used to assess the strengths of the tested promoters in LL3 (Additional file 1: Figure S5).

### Characterization of the selected promoters via RT-qPCR and GFP fluorescence measurement

**Fig. 5:**
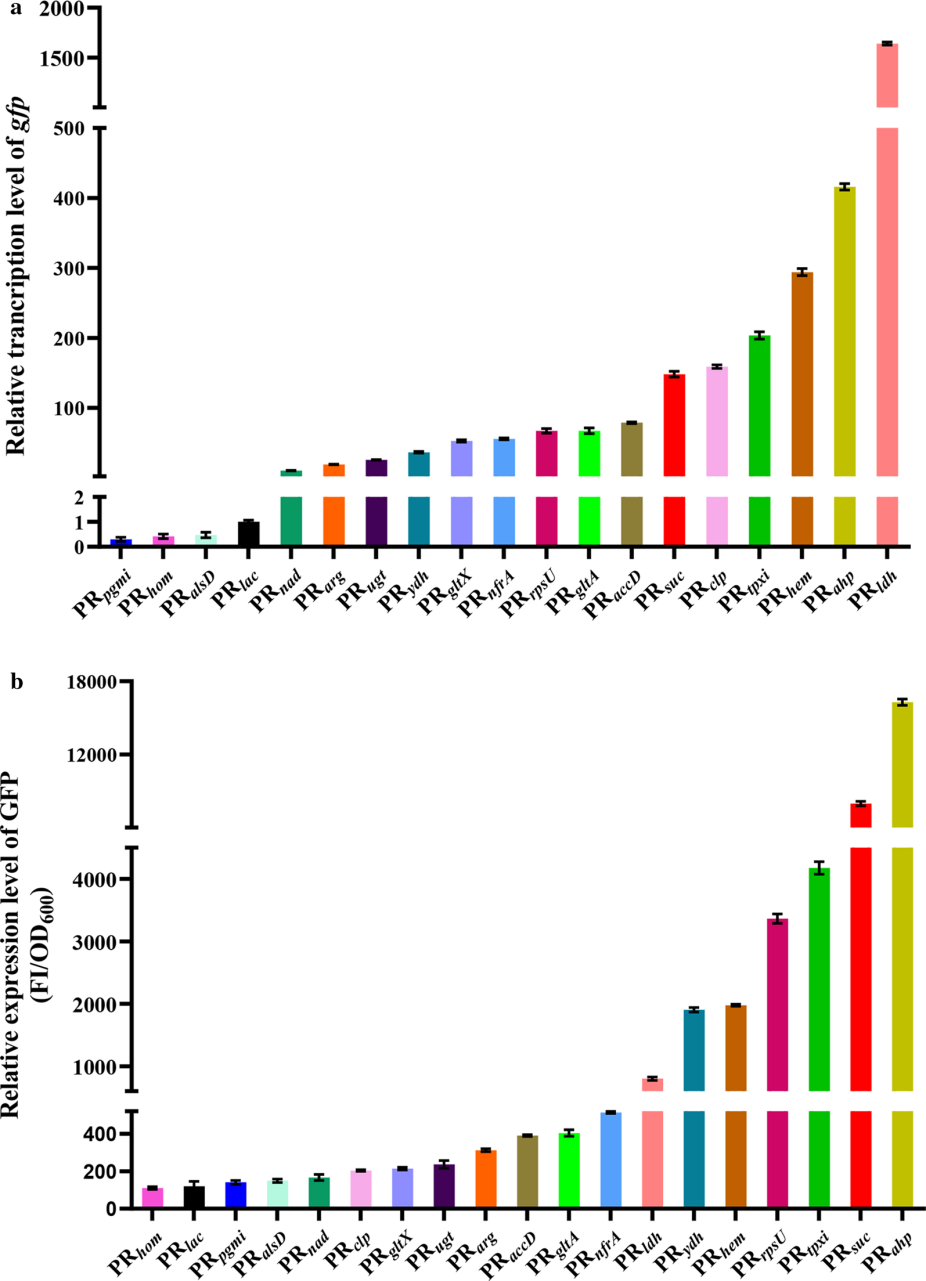
Characterization of the strengths of the selected endogenous promoters using reporter gene assays in LL3. **a** Transcriptional level of *gfp* gene quantified via qPCR under the control of different promoters (*rpsU* gene was used as internal standard; the transcriptional level of *gfp* gene controlled by *lac* promoter was set as 1); **b** the relative fluorescence intensity of GFP (FI/OD_600_) under the control of different promoters. Values denote mean ± SD of triplicates

As shown in Fig. 5a, the relative transcriptional levels of the candidate promoters measured with reporter gene vectors were PR_*ldh*_, PR_*ahp*_, PR_*hem*_, PR_*tpxi*_, PR_*clp*_, PR_*suc*_, PR_*accD*_, PR_*glt*A_, PR_*rpsu*_, PR_*nfrA*_, PR_*gltX*_, PR_*ydh*_, PR_*ugt*_, PR_*arg*_, PR_*nad*_, PR_*lac*_, PR_*alsD*_, PR_*hom*_ and PR_*pgmi*_ in a descending order, which were inconsistent with the strengths of the promoters shown by the FPKM values (Additional file 1: Table S3), with similar results reported in a previous study [23]. We speculate that the transcription of a gene on chromosome may be affected and regulated by flanking genes and regulatory sequences. However, this interference could be eliminated if a promoter is inserted into a plasmid.

To better evaluate these endogenous promoters, the relative fluorescence intensities of GFP was measured. Among the 18 endogenous promoters, PR_*ahp*_, PR_*suc*_ and PR_*tpxi*_ showed superior production capacity of GFP, followed by PR_*rpsU*_, PR_*hem*_ and PR_*ydh*_ (Fig. 5b). However, the first six promoters were PR_*ldh*_, PR_*ahp*_, PR_*hem*_, PR_*tpxi*_, PR_*clp*_ and PR_*suc*_ from high to low at the transcriptional levels (Fig. 5a). The different RBSs located upstream of the promoters may affect the translational initiation efficiencies of mRNA corresponding to GFP, leading to the different trends between the transcriptional level and production capacity of GFP.

### Substitution of the native srfA promoter further enhanced surfactin production

**Fig. 6:**
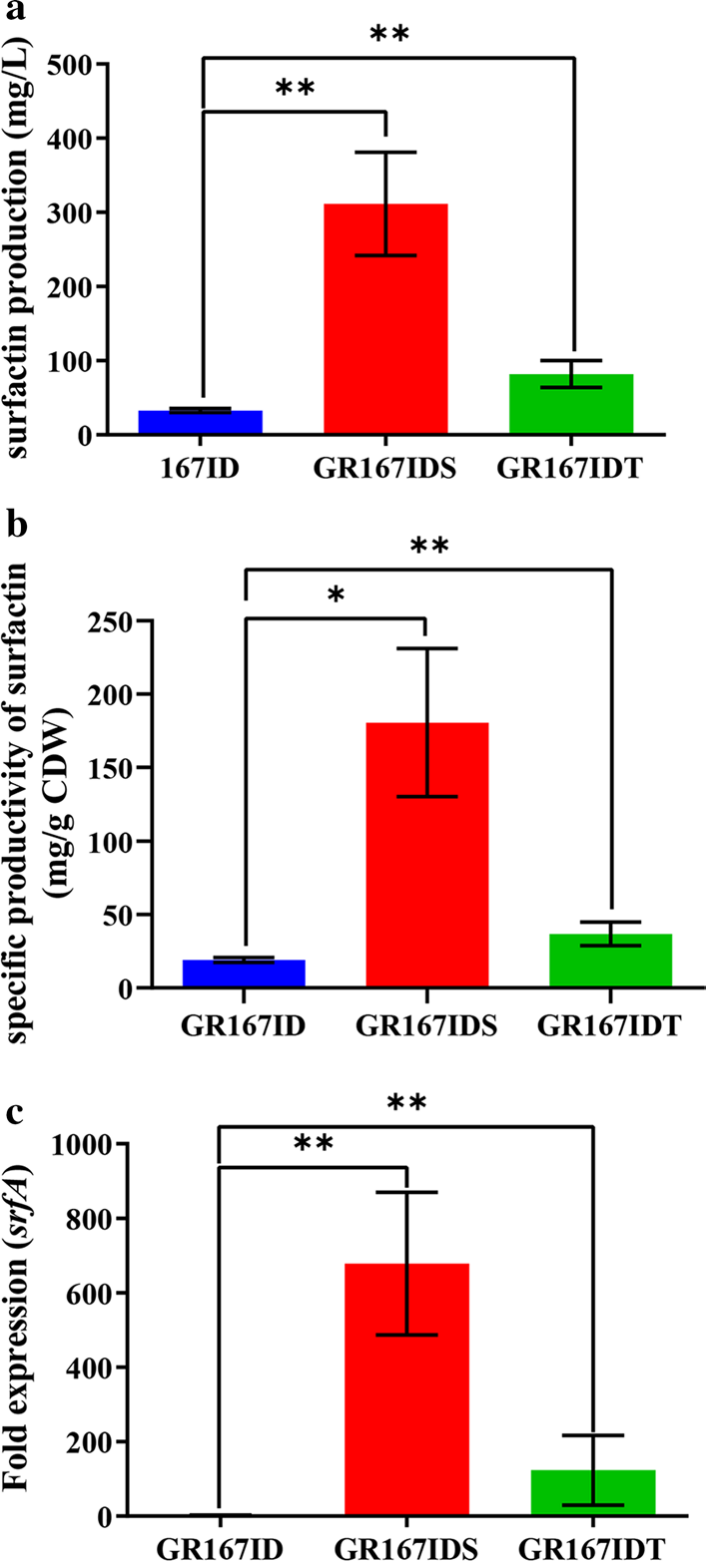
Surfactin production by GR167ID, GR167IDS and GR167IDT, and transcriptional levels of *srfA* operon in the strains. **a** Surfactin production; **b** specific productivity of surfactin (mg/g CDW, the ratio of surfactin production to cell dry weight); **c** transcriptional levels of *srfA* operon quantified via qPCR (the transcriptional level of *srfA* operon in GR167ID was set as 1). Values denote mean ± SD of triplicates (*P < 0.05, **P < 0.01)

Considering the heterologous expression of *srfA* is challenging for which large genetic sequence (over 25 kb), substitution of the native *srfA* promoter by strong promoters is considered more beneficial for enhanced transcription of *srfA* operon [15, 16, 20]. In this study, two promoters PR_*suc*_ and PR_*tpxi*_ with better transcription and expression levels were integrated into upstream of the *srfA* operon in GR167ID, generating mutant strains GR167IDS and GR167IDT, respectively. The nucleotide sequences of the two selected promoters are shown in supplementary material. As expected, both the surfactin production and specific productivity exhibited a significant elevation (Fig. 6a, b). In particular, the PR_*suc*_ promoter-substituted strain GR167IDS produced 311.35 mg/L surfactin, which was about 9.5-fold higher than that of GR167ID (Fig. 6a). Meanwhile, the transcriptional level of *srfA* in GR167IDS was 678-fold higher than that in GR167ID (Fig. 6c). The melting curves of *srfA* and its internal standard gene *rpsU* indicated the absence of non-specific products (Additional file 1: Figure S6).

The chassis strain GR167 does not differ in the surfactin yield compared to NK-ΔLP, however, the replacement of *srfA* promoter with PR_*suc*_ promoter in GR167ID significantly increased the surfactin yield of the promoter-substituted strain GR167IDS compared with other modifications (genome reduction and blocking of competitive pathways). Interestingly, PR_*suc*_ was substituted for *srfA* promoter in NK-ΔLP to generate the mutant strain NK-ΔLPS, with a surfactin yield of 180.88 ± 2.87 mg/L (approximately 1.7-fold lower than that of GR167IDS). Therefore, genome reduction may contribute to the improvement of overall cellular metabolic activity, resulting in high-performance production of surfactin in GR167IDS.

## Conclusions

In summary, a genome-reduced strain GR167 was constructed by deleting some non-essential genes accounting for ~ 4.18% of the LL3 genome and outcompeted the parental strain in several physiological traits assessed. GR167IDS, obtained from GR167 by promoter substitution, showed a 10.4-fold improvement in the titer of surfactin compared to GR167. The current results suggest that genome reduction in combination with promoter engineering may be a feasible strategy for the development of microbial cell factories capable of efficiently producing bacterial secondary metabolites.

## Methods

### Bacterial strains, media, and culture conditions

*Escherichia coli* DH5α was employed for plasmid construction and propagation. For the subsequent successful electroporation of *B. amyloliquefaciens* strains, the *E. coli* JM110 was used as intermediate host to demethylate the desirable plasmids from *E. coli* DH5α. *E. coli* strains were incubated at 37 °C in Luria–Bertani (LB) broth. *B. amyloliquefaciens* LL3 was deposited in the China Center for Type Culture Collection (CCTCC) (accession number: CCTCC M 208109). *B. amyloliquefaciens* NK-1 was employed as the parental strain for genome reduction. GR167 was used as the starting strain for engineered high-yielding surfactin producing mutants. M9 medium, which contains 3.4 g/L Na_2_HPO·12H_2_O, 0.6 g/L KH_2_PO_4_, 0.1 g/L NaCl, 0.2 g/L NH_4_Cl supplemented with 20 g/L glucose, 50 mg/L tryptophan and 200 mM MOPS, was used for assessing growth of relevant strains. For lipopeptide surfactin production, *B. amyloliquefaciens* was incubated at 30 °C and 180 rpm for 48 h in Landy medium [41]. When appropriate, media were supplemented with ampicillin (Ap; 100 μg/mL), chloramphenicol (Cm; 5 μg/mL) or 5-fluorouracil (5-FU; 1.3 mM).

### Plasmid and strain construction

To construct the gene deletion vectors, the temperature-sensitive plasmid pKSU with an *upp* expression cassette was used [25]. The upstream and downstream fragments of the deleted genomic regions were amplified by PCR and then the two fragments were joined by overlap PCR. The generated fragment was ligated into pKSU via homologous recombination, to generate the gene deletion vectors. Introduction of plasmid into *B. amyloliquefaciens* was carried out using an optimized high osmolarity electroporation method [36]. To carry out multiple gene deletions on a single strain, a marker-less gene deletion method was used to construct the gene knockout mutants [24]. All the constructed plasmids and mutant strains were validated by PCR detection and DNA sequencing. All plasmids, strains, and primers used in this study are listed in Table 2, Additional file 1: Tables S4, S5, respectively.

**Table 2:** Strains used in this study

Strains	Relative characteristics	Source
*B. amyloliquefaciens*		
GR01	LL3 derivative, Δ*upp*	[24]
GR07 (NK-1)	GR01 ΔG0, 0.18% reduction of genome	[25]
GR22	GR07 ΔG1, 0.55% reduction of genome	This work
GR46	GR22 ΔG2, 1.15% reduction of genome	This work
GR94	GR46 ΔG3, 2.36% reduction of genome	This work
GR134	GR94 ΔG4, 3.36% reduction of genome	This work
GR164	GR134 ΔG5, 4.11% reduction of genome	This work
GR167	GR164 ΔG6, 4.18% reduction of genome	This work
NK-ΔLP	NK-1 derivative, Δ*pgsBCA*	[37]
GR167I	GR167 derivative, Δ*itu* cluster	This work
GR167D	GR167 derivative, Δ*fen* cluster	This work
GR167ID	GR167 derivative, Δ*itu* Δ*fen* clusters	This work
LL3-PR_*lac*_	LL3 derivative, containing plasmid pHT-PR_*lac*_-*gfp*	This work
LL3-PR_*ugt*_	LL3 derivative, containing plasmid pHT-PR_*ugt*_-*gfp*	This work
LL3-PR_*suc*_	LL3 derivative, containing plasmid pHT-PR_*suc*_-*gfp*	This work
LL3-PR_*ydh*_	LL3 derivative, containing plasmid pHT-PR_*ydh*_-*gfp*	This work
LL3-PR_*accD*_	LL3 derivative, containing plasmid pHT-PR_*accD*_-*gfp*	This work
LL3-PR_*clp*_	LL3 derivative, containing plasmid pHT-PR_*clp*_-*gfp*	This work
LL3-PR_*tpxi*_	LL3 derivative, containing plasmid pHT-PR_*tpxi*_-*gfp*	This work
LL3-PR_*gltX*_	LL3 derivative, containing plasmid pHT-PR_*gltX*_-*gfp*	This work
LL3-PR_*nad*_	LL3 derivative, containing plasmid pHT-PR_*nad*_-*gfp*	This work
LL3-PR_*arg*_	LL3 derivative, containing plasmid pHT-PR_*arg*_-*gfp*	This work
LL3-PR_*gltA*_	LL3 derivative, containing plasmid pHT-PR_*gltA*_-*gfp*	This work
LL3-PR_*ahp*_	LL3 derivative, containing plasmid pHT-PR_*ahp*_-*gfp*	This work
LL3-PR_*nrfA*_	LL3 derivative, containing plasmid pHT-PR_*nrfA*_-*gfp*	This work
LL3-PR_*pgmi*_	LL3 derivative, containing plasmid pHT-PR_*pgmi*_-*gfp*	This work
LL3-PR_*hom*_	LL3 derivative, containing plasmid pHT-PR_*hom*_-*gfp*	This work
LL3-PR_*hem*_	LL3 derivative, containing plasmid pHT-PR_*hem*_-*gfp*	This work
LL3-PR_*ldh*_	LL3 derivative, containing plasmid pHT-PR_*ldh*_-*gfp*	This work
LL3-PR_*rpsU*_	LL3 derivative, containing plasmid pHT-PR_*rpsU*_-*gfp*	This work
LL3-PR_*alsD*_	LL3 derivative, containing plasmid pHT-PR_*alsD*_-*gfp*	This work
GR167IDS	GR167ID derivative with its native *srf* promoter replaced by PR_*suc*_	This work
GR167IDT	GR167ID derivative with its native *srf* promoter replaced by PR_*tpxi*_	This work
NK-ΔLPS	NK-ΔLP derivative with its native *srf* promoter replaced by PR_*suc*_	This work
*E. coli* strains		
DH5α	*supE44* Δ*lacU169*(_*80 lacZ*ΔM15) *recA1 endA1 hsdR17*(r_K_^−^ m_K_^+^) *thi-1gyrA relA1* F^−^ Δ(*lacZYA-argF*)	Transgene
JM110	F^−^*dam-*13::Tn9 (Cam^r^) *dcm*-6 *hsdR2* (r_k_^−^m_k_^+^) *leuB6 hisG4 thi-1 araC14 lacY1 galK2 galT22 xylA5 mtl-1 rpsL136* (Str^r^) *fhuA31 tsx-8 glnV*44 *mcrA mcrB*1	Fermentas

### Physiological traits assessment

Growth profiles of GR167 and NK-1 were measured in both M9 mineral medium and LB medium. Overnight cultures (1 mL) were inoculated into 100 mL LB or M9 medium in 500-mL flasks and then incubated for 20 h at 37 °C and 180 rpm. To determine the bacterial growth status, the OD_600_ was monitored every 2 h using a UV-1800 spectrophotometer (Shimadzu, Kyoto, Japan).

The metabolic phenotypic analyses were performed with a Biolog GEN Ш MicroPlate™ using a phenotype microarray system (Biolog Inc., California, USA) according to the manufacturer’s instructions. The bacterial cells on the solid medium surface were collected by cotton swab, dissolved into the inoculating fluid IF-B, and then the cell density was adjusted to a range of 80–86% turbidity. Subsequently, 100 μL of bacterial suspensions were pipetted into the Biolog GEN Ш plates with different substrates. After the samples were incubated at 33 °C for 48 h, the absorbance at 590 nm was measured with the Biolog reader and the test data were analyzed by the Biolog system.

Electro-competent cells of GR167 and NK-1 (2 × 10^10^ CFU/mL) were prepared according to previous methods [36]. Subsequently, approximately 100 ng of plasmid pHT01 was absorbed by 100 μL of electro-competent cells via electroporation. After 3 h of recover at 37 °C and 180 rpm, the mixture was spread on LB agar plates supplemented with 5 μg/mL Cm. To eliminate the growth-rate bias of different strains, transformation efficiency was calculated by normalizing the colony number of bacteria transformed with plasmid pHT01 against the colony number of bacteria transformed without plasmid DNA.

Cells were cultured in LB medium at 37 °C for 18 h. The intracellular cofactors NADPH and NADP^+^ were extracted and quantified by enzymatic methods [41] using an EnzyChrom™ assay kit (BioAssay Systems, USA) according to the manufacturer’s protocols.

The heterologous protein productivity was determined by introducing plasmid pHT-P_43_-*gfp* into GR167 and NK-1. The detailed protocols for strain cultivation and fluorescence intensity measurement refer to our previous work [9]. The relative fluorescence intensity was normalized against per OD_600_ of whole cells. The fluorescence signal of NK-1 harboring pHT01 was set as background and was subtracted from overall fluorescence.

### RNA-seq, promoter prediction, and construction of reporter gene vectors

RNA-seq analyses of LL3 were carried out according to our previous methods [32]. The expression levels of the predicted genes were quantified as the FPKM value [42]. The upstream regions of genes with different FPKM values were submitted online (http://www.fruitfly.org/seq_tools/promoter) for promoter prediction.

Furthermore, each promoter sequence plus its native RBS and *gfp* gene were amplified by PCR from the LL3 genome and pHT-P_43_-*gfp*, respectively. Subsequently, 3′-end of a promoter sequence was fused with 5′-end of *gfp* gene and the fusion fragment was inserted into plasmid pHT01, to generate reporter gene vector pHT-PR_*x*_-*gfp* for promoter strength characterization (Additional file 1: Figure S5). Moreover, a control vector pHT-PR_*lac*_-*gfp* with *gfp* expression driven by *lac* promoter was similarly constructed with pHT01.

### Total RNA extraction, qPCR analyses, and GFP fluorescence measurement of reporter gene vectors

An appropriate number of cells from LB or Landy cultures were collected to isolate total RNA with the RNApure Bacteria kit (DNase I) (Cwbio, Beijing, China). Afterwards, complementary DNA (cDNA) was prepared with approximately 0.5 μg total RNA as template employing the HiScript® II Q RT SuperMix (Vazyme). To determine the transcriptional strength of relevant genes, qPCR analysis was carried out with ChamQ Universal SYBR qPCR Master Mix (Vazyme) and cDNA as the template. The relative gene transcription levels were calculated against that of *rpsU* gene as the internal standard using the 2^−ΔΔCt^ method [43, 44]. The relative transcriptional activity of a promoter was normalized against that of *lac* promoter. In addition, GFP fluorescence measurement was performed as described previously [9].

### Surfactin isolation and HPLC–MS analyses

All tested strains were cultured aerobically at 180 rpm in the Landy medium for 48 h. The culture supernatant and bacterial cell were separated by centrifugation at 4 °C and 14,000 rpm for 20 min. Subsequently, the bacterial cell was lyophilized for 24 h and weighed to obtain the cell dry weight (CDW). The supernatant was acidified to pH 2.0 with 6 M HCl and precipitated overnight at 4 °C. The precipitate formed was harvested by centrifugation and resuspended with 100 mL methyl alcohol. After which, 1 M NaOH was added to adjust the pH to 7.0 and further incubated for 48 h at 180 rpm and 37 °C. The supernatant containing surfactin extract was collected by centrifugation. The recovery and purification of surfactin homologues was performed as previously described [17]. Prior to HPLC–MS analysis, the supernatant was concentrated through a vacuum rotary evaporator and filtered via a 0.22-μm filter. Surfactin was analyzed and quantified by HPLC–MS equipped with a C18 column (Innoval ODS-2, 250 mm × 4.6 mm × 5 μm, Phenomenex, USA) using a validated method described previously [17, 42]. The extracted surfactin samples (20 μL) were injected into the HPLC–MS system with a mobile phase consisting of acetonitrile and water (55:45, v/v) at a flow rate of 0.8 mL/min. Surfactin was detected at 210 nm.

## Supplementary Information

**Additional file 1.** Additional figures and tables.

## Data Availability

All data generated or analyzed during the current study are included in this published article and its Additional file.
