# Supplementary material for: Engineering of a genome-reduced strain Bacillus amyloliquefaciens for enhancing surfactin production
Source: Microb Cell Fact. 2020 Dec 7;19:223. doi: 10.1186/s12934-020-01485-z (PMC7720510; doi:10.1186/s12934-020-01485-z)
Supplement: Supplementary file 1 — Additional file 1. Additional figures and tables. [file 12934_2020_1485_MOESM1_ESM.docx]

**Additional material**

Engineering of a genome-reduced strain *Bacillus amyloliquefaciens* for enhancing surfactin production

Fang Zhang,^1^ Kaiyue Huo,^1^ Xingyi Song,^1^ Yufen Quan,^1^ Shufang Wang,^1^ Zhiling Zhang,^2,3^* Weixia Gao,^4^* Chao Yang^1^*

^1^Key Laboratory of Molecular Microbiology and Technology for Ministry of Education, Key Laboratory of Bioactive Materials for Ministry of Education, College of Life Sciences, Nankai University, Tianjin, China

^2^Department of Maxillofacial Radiology, Tianjin Stomatological Hospital, School of Medicine, Nankai University, Tianjin 300041,China

^3^Tianjin Key Laboratory of Oral and Maxillofacial Function Reconstruction, Tianjin 300041,China

^4^MOE Key Laboratory of Industrial Fermentation Microbiology, College of Biotechnology, Tianjin University of Science and Technology, Tianjin, China

*Correspondence to:

Zhiling Zhang (Tel./Fax: +86 22 59080620, E-mail: zhilingzhang@nankai.edu.cn)

Weixia Gao (Tel./Fax: +86 22 60601566, E-mail: gaoweixia@tust.edu.cn)

Chao Yang (Tel./Fax: +86 22 23503866, E-mail: yangc20119@nankai.edu.cn)

**
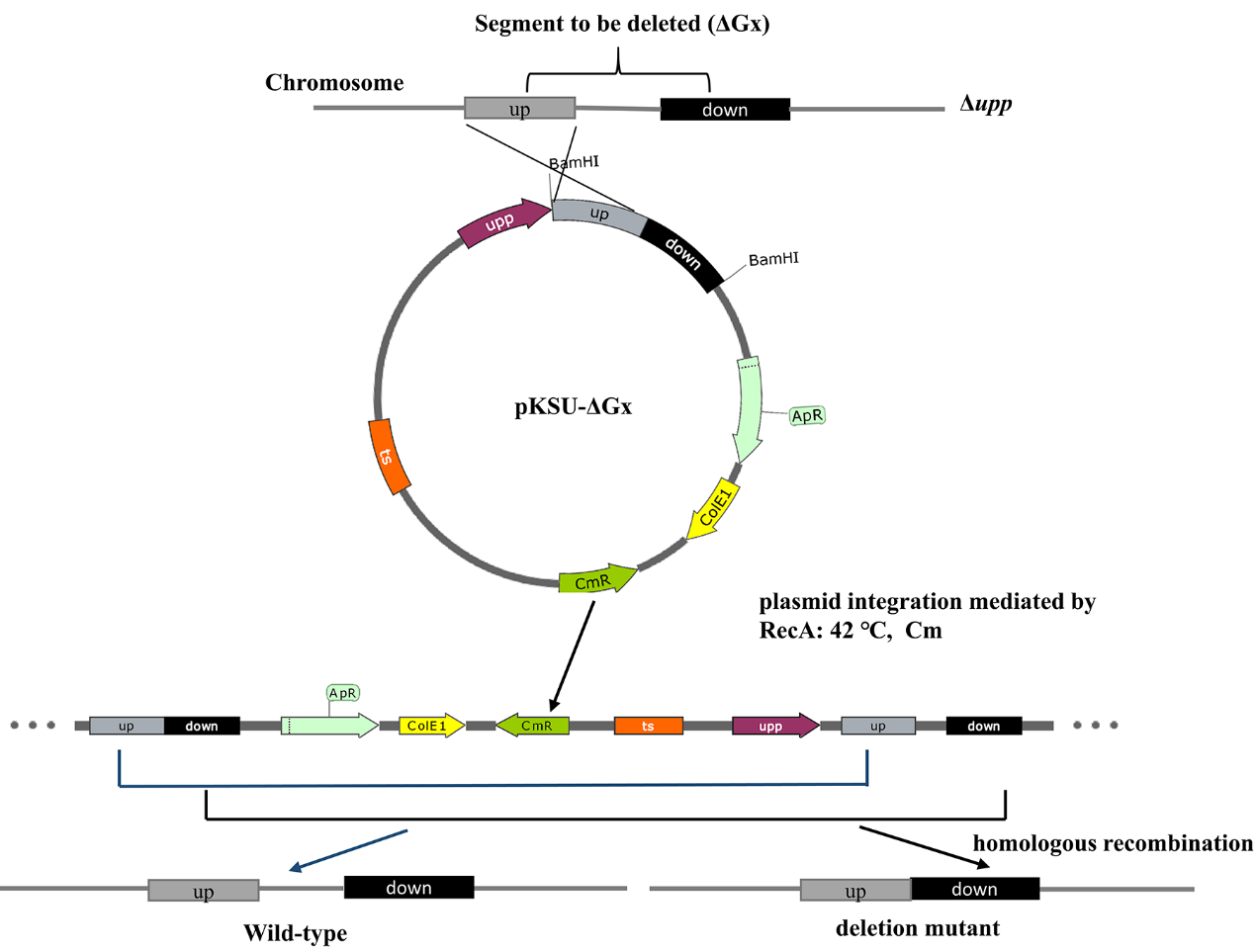
**

**Figure S1** Schematic diagram for scarless deletion of large-segments in the *B. amyloliquefaciens*. Ap^R^, ampicillin resistance gene; Cm^R^, chloramphenicol resistance gene.

**
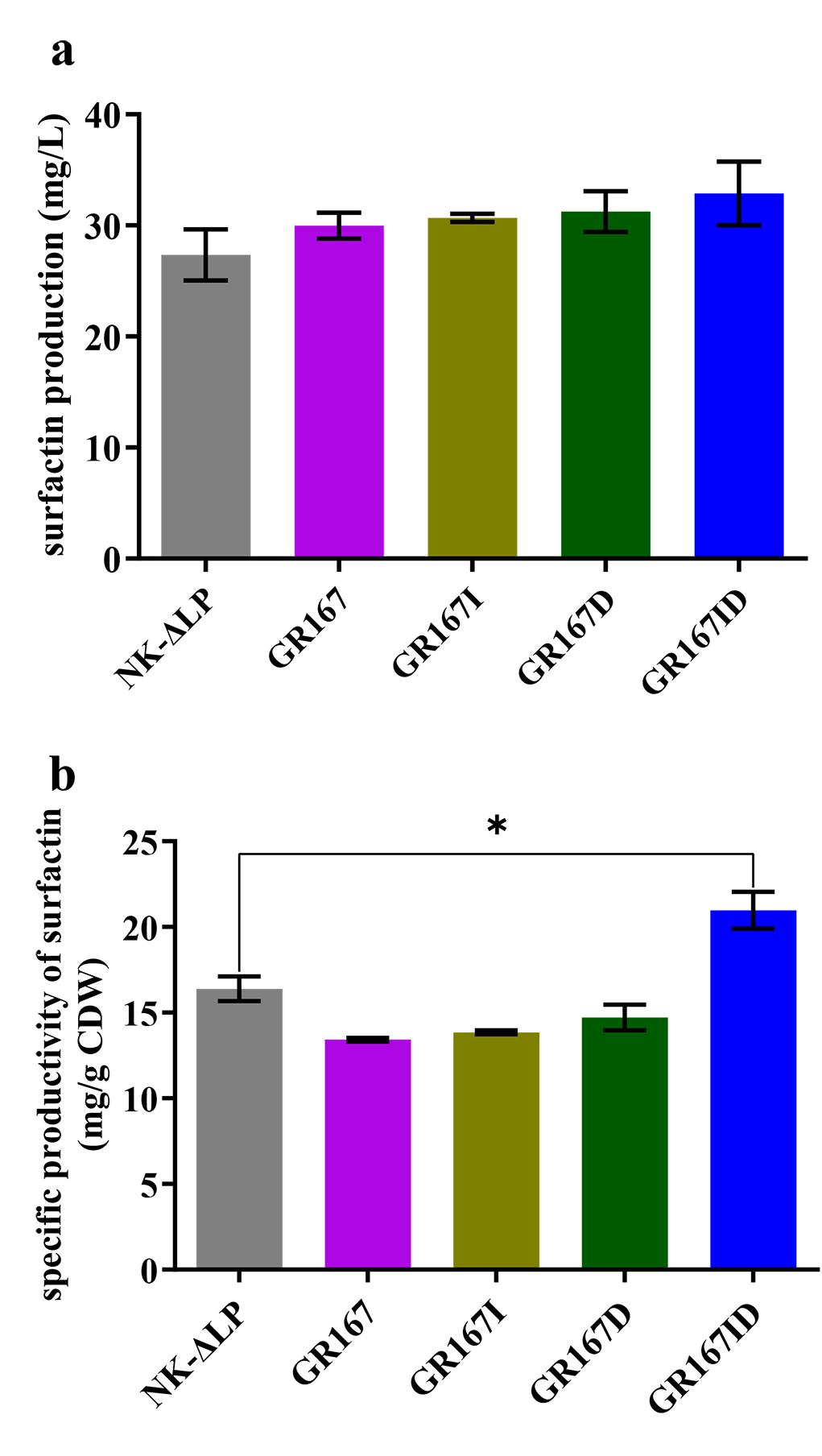
**

**Figure S2** Surfactin production by GR167 and its derivatives. **a** Surfactin production; **b** specific productivity of surfactin (mg/g CDW, the ratio of surfactant production to cell dry weight). To accumulate surfactin, the strains were incubated in Landy medium for 48 h at 30 °C and 180 rpm. NK-ΔLP was used as a control. Values denote mean ± SD of triplicates (**P* < 0.05)


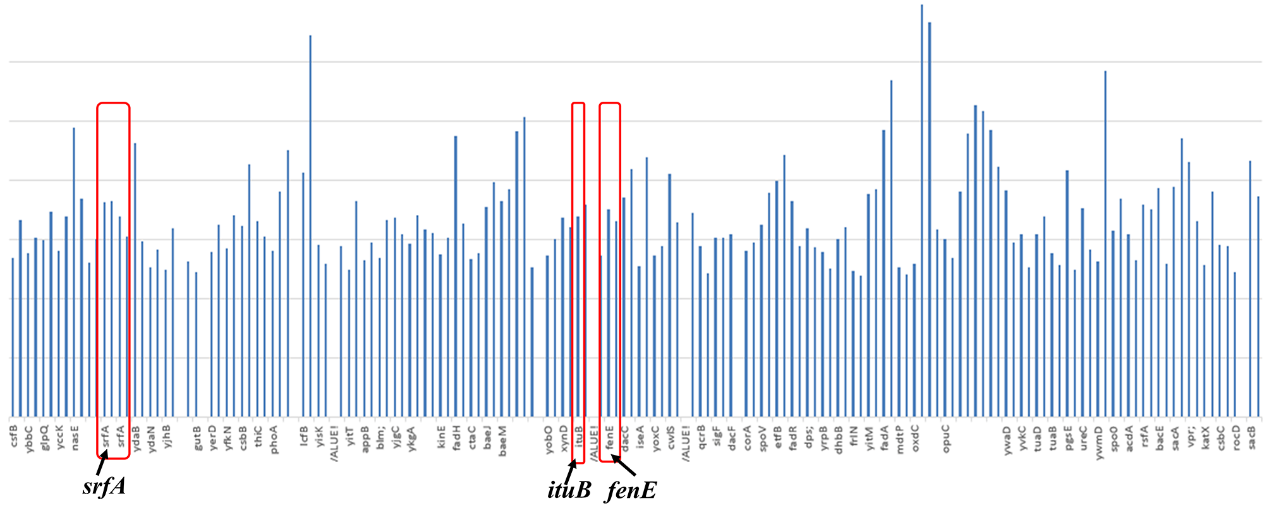


**Figure S3** The up-regulated genes in the transcriptome of NK-ΔLP compared with that of the *B. amyloliquefaciens* LL3. The *srfA*, *ituB* and *fenE* genes are responsible for the biosynthesis of surfactin, iturin and fengycin, respectively.


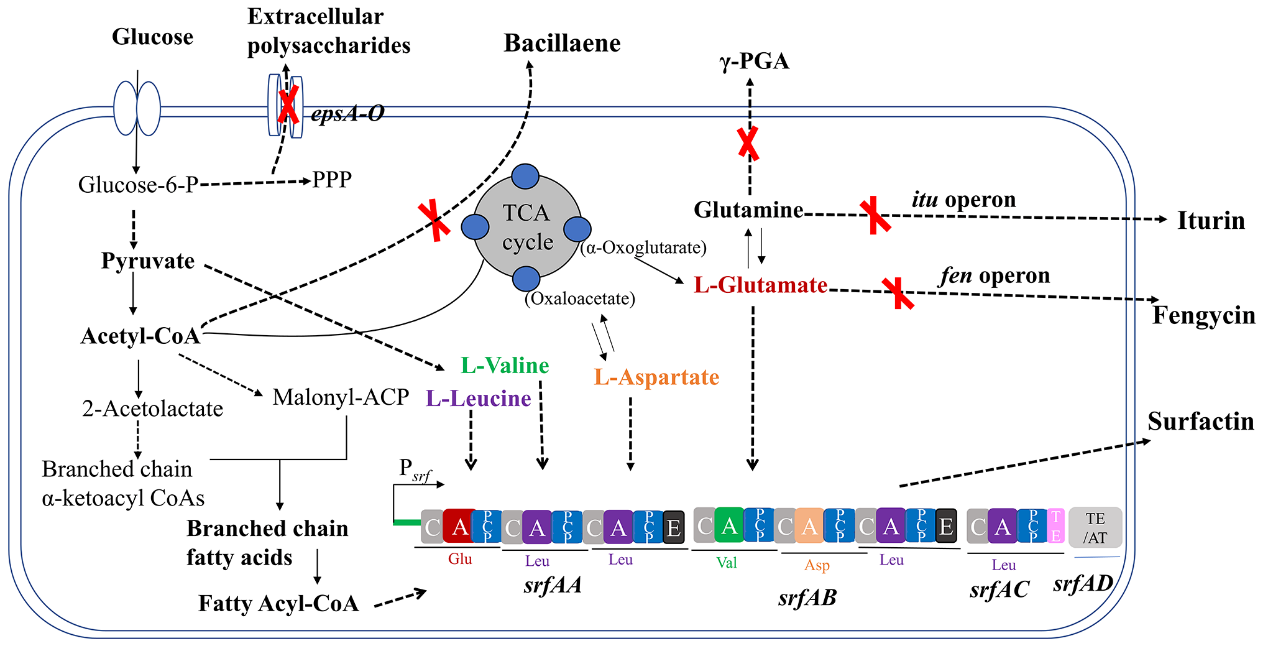


**Figure S4** Schematic of surfactin biosynthesis pathway in *Bacillus.* sp. The four amino acids (shown in red, orange, green and purple words) are assembled onto fatty acyl-CoA via NRPS encoded by *srfA* operon. The domains of adenylation domain (A), peptidyl carrier protein (PCP), and condensation (C) are used for selecting and activating substrates, carrying aminoacyl-adenylate, and forming peptide bonds, respectively. Epimerization (E) and thioesterase (TE) are responsible for the stereochemical conversion and the relase of products. Glu, Glutamate; Leu, Leucine; Val, Valin; Asp, Aspartate. The X marked in red indicate the blocked pathways during genome reduction or the optimized pathway


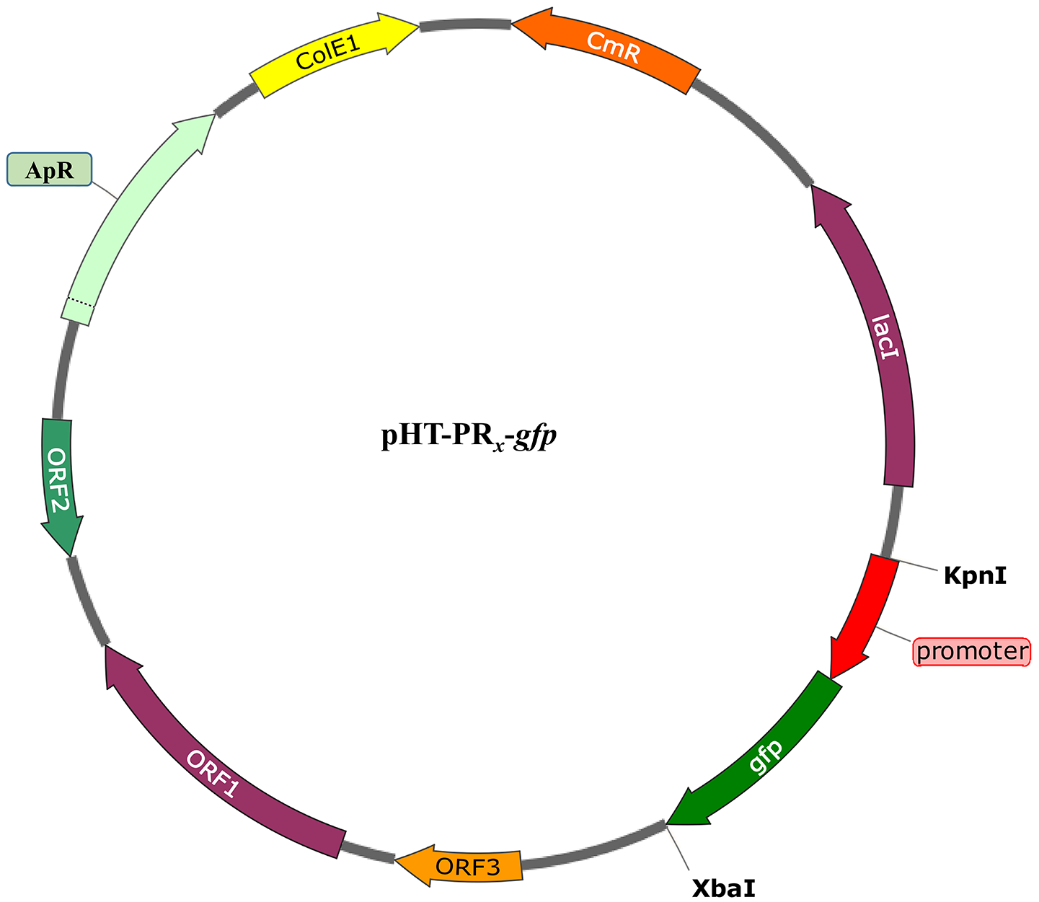


**Figure S5** Map of recombinant vectors harboring respectively the eighteen endogenous promoters (PR*_ugt_*, PR*_suc_*, PR*_ydh_*, PR*_accD_*, PR*_clp_*, PR*_tpxi_*, PR*_gltX_*, PR*_nad_*, PR*_arg_*, PR*_gltA_*, PR*_ahp_*, PR*_nrfA_*, PR*_pgmi_*, PR*_hom_*, PR*_hem_*, PR*_ldh_*, PR*_rpsU_*, PR*_alsD_*) and *lac* promoter. *gfp*, reporter gene; Ap^R^, ampicillin resistance gene; Cm^R^, chloramphenicol resistance gene.

**
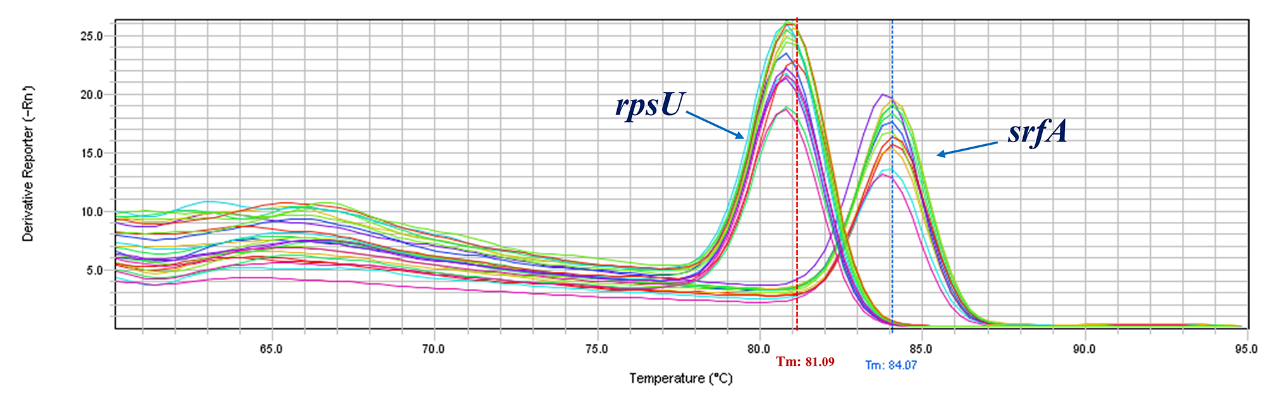
**

**Figure S6** Melting curves of *srfA* and *rpsU* gene in GR167ID, GR167IDS and GR167IDT.

**Table S1** **Regions deleted during the construction of genome-reduced strain GR167**

| **Deleted regions** | **Note** | **Position (start-end)** | **Amount of DNA removed (kb)** | **Content of G+C (%)** |
| --- | --- | --- | --- | --- |
| *upp* ^a^ | uracil phosphoribosyltransferase | 3597040-3597659 | 0.66 | / |
| G0 (pMC1) ^b^ | endogenous plasmid | / | 6.76 | / |
| G1 (*eps* cluster) | extracellular polysaccharide synthase | 3347296-3363008 | 15.71 | / |
| G2 (prophage 1) | phage-associated protein | 2247906-2271732 | 23.84 | 38 |
| G3 (partial *bae* cluster) | bacillaene synthesis | 1745467-1792824 | 47.36 | / |
| G4 (prophage 2) | phage-associated protein | 850951-891433 | 40.48 | 41 |
| G5 (prophage 3) | phage-associated protein | 2159768-2189833 | 30.07 | 36 |
| G6 (*pgsBCA* cluster) | γ-polyglutamate synthetase | 3506811-3509823 | 3.01 | / |

a *upp* deleted by Zhang *et al.* (2014)

b endogenous plasmid deleted by Feng *et al*. (2013)

**Table S2** **Gene annotation of** **streamlined genomic regions in *B. amyloliquefaciens* LL3**

| Deletion units | Gene name | Position (start-end) | Product |
| --- | --- | --- | --- |
| *upp* | *upp* | [3597040..3597759](-) | uracil phosphoribosyltransferase |
| G0 | pMC1 | / | endogenous plasmid |
| G1 | *epsO* | [3347296..3348258](-) | polysaccharide pyruvyl transferasefamily protein |
|  | *epsN* | [3348237..3349409](-) | aminotransferase class I/II-fold pyridoxal phosphate-dependent enzyme |
|  | *epsM* | [3350058..3350666](-) | sugar transferase |
|  | *epsL* | [3350663..3352180](-) | MATE family efflux transporter |
|  | *epsK* | [3352177..3353211](-) | glycosyltransferase |
|  | *epsI* | [3353208..3354284](-) | polysaccharide pyruvyl transferase family protein |
|  | *epsH* | [3354289..3355326](-) | glycosyltransferase family 2 protein |
|  | *epsG* | [3355345..3356448](-) | EpsG family protein |
|  | *epsF* | [3356452..3357588](-) | glycosyltransferase family 1 protein |
|  | *epsE* | [3357581..3358423](-) | glycosyltransferase family 2 protein |
|  | *epsD* | [3358420..3359559](-) | glycosyltransferase family 4 protein |
|  | *epsC* | [3359575..3361368](-) | polysaccharide biosynthesis protein |
|  | *epsB* | [3361615..3362295](-) | CpsD/ CapB family tyrosine-protein kinase |
|  | *epsA* | [3362301..3363008](-) | hypothetical protein |
| G2 | *yomK* | [2247972..2248382](+) | hypothetical protein |
|  | *puta* | [2248454..2249080](+) | hypothetical protein |
|  | unknown | [2249127..2256233](+) | phage tail family protein |
|  | *yomG* | [2257054..2259687](+) | hypothetical protein |
|  | unknown | [2259913..2260080](+) | hypothetical protein |
|  | *yomF* | [2260174..2260989](+) | hypothetical protein |
|  | *phi1* | [2261003..2263552](+) | hypothetical protein |
|  | *xlyA* | [2263725..>2264183](+) | N-acetylmuramoyl-L-alanine amidase |
|  | unknown | [<2264604..>2264828](+) | N-acetylmuramoyl-L-alanine amidase |
|  | unknown | [<2264829..2265107](+) | N-acetylmuramoyl-L-alanine amidase family protein |
|  | unknown | [2265221..2265613](+) | hypothetical protein |
|  | unknown | [2265634..2265885](+) | phage holin |
|  | unknown | [2266223..2266528](+) | hypothetical protein |
|  | *RapI* | [2266680..2267840](+) | etratricopeptide repeat protein |
|  | *uvrX* | [2268004..2268618](-) | DNA polymerase |
|  | unknow | [2268659..2270470](-) | maturase |
|  | *uvrX* | [2271022..2271729](-) | hypothetical protein |
| G3 | unknown | [1745483..1746160](+) | MBL fold metallo-hydrolase |
|  | *baeC* | [1746465..1747334](+) | ACP S-malonyltransferase |
|  | *baeD* | [1747469..1748443](+) | acyltransferase domain-containing protein |
|  | *baeE* | [1748445..1750682](+) | ACP S-malonyltransferase |
|  | *acpK* | [1750750..1750998](+) | acyl carrier protein |
|  | *baeG* | [1751050..1752312](+) | hydroxymethylglutaryl-CoA synthase family  protein |
|  | *baeH* | [1752309..1753082](+) | enoyl-CoA hydratase/isomerase |
|  | *baeI* | [1753092..1753841](+) | enoyl-CoA hydratase/isomerase family protein |
|  | *baeJ* | [1753881..1768832](+) | non-ribosomal peptide synthetase |
|  | *baeL* | [1768834..1782264](+) | SDR family NAD(P)-dependent oxidoreductase |
|  | *baeL* | [1782282..1792823](+) | SDR family NAD(P)-dependent oxidoreductase |
| G4 | *csbB* | [851154..852134](+) | glycosyltransferase family 2 protein |
|  | *yfhO* | [852171..854756](+) | YfhO family protein |
|  | *yfhP* | [854753..855736](-) | metal-dependent hydrolase |
|  | *xerC* | [855957..857105](-) | site-specific integrase |
|  | unknown | [857173..857637](-) | ImmA/IrrE family metallo-endopeptidase |
|  | unknown | [857652..857957](-) | helix-turn-helix transcriptional regulato |
|  | unknown | [858230..858430](+) | helix-turn-helix transcriptional regulator |
|  | unknown | [858417..858689](+) | hypothetical protein |
|  | unknown | [858739..858888](+) | hypothetical protein |
|  | *yqaH* | [858885..859166] (+) | hypothetical protein |
|  | unknown | [859153..85946](+) | hypothetical protein |
|  | *yoqD* | [859452..860099](+) | Rha family transcriptional regulator |
|  | unknown | [860112..860957](+) | hypothetical protein |
|  | unknown | [861119..861487](+) | hypothetical protein |
|  | *repO* | [861499..862272](+) | hypothetical protein |
|  | unknown | [862265..862621](+) | hypothetical protein |
|  | *dnaB* | [862622..863950](+) | replicative DNA helicase |
|  | unknown | [863940..864152](+) | hypothetical protein |
|  | unknown | [864149..864349](+) | hypothetical protein |
|  | unknown | [864448..864906](+) | ArpU family transcriptional regulator |
|  | unknown | [865239..865451](+) | cell division protein FtsK |
|  | unknown | [865789..866403](+) | hypothetical protein |
|  | unknown | [866416..866616](+) | hypothetical protein |
|  | unknown | [866648..866962](+) | HNH endonuclease |
|  | unknown | [867191..867646](+) | phage terminase small subunit P27 family |
|  | *gp02* | [867636..869426](+) | terminase large subunit |
|  | *gp03* | [869438..870658](+) | phage portal protein |
|  | *ATP-* | [870633..871355](+) | Clp protease ClpP |
|  | *gp36* | [871352..872557](+) | phage major capsid protein |
|  | unknown | [872571..872885](+) | phage head-tail connector protein |
|  | *phag* | [872892..873221](+) | phage head closure protein |
|  | *gp10* | [873218..873646](+) | HK97 gp10 family phage protein |
|  | unknown | [873643..874032](+) | hypothetical protein |
|  | majo | [874091..874666](+) | tail protein |
|  | unknown | [874742..875062](+) | hypothetical protein |
|  | *yqbO* | [875245..881001](+) | phage tail tape measure protein |
|  | *gp17* | [881004..881843](+) | phage tail family protein |
|  | *gp18* | [881853..883007](+) | phage tail protein |
|  | phag | [883000..88330](+) | hypothetical protein |
|  | unknown | [883500..884315](+) | SGNH/GDSL hydrolase family protein |
|  | *gp24* | [884331..885617](+) | BppU family phage baseplate upper protein |
|  | unknown | [885618..885953](+) | DUF2977 domain-containing protein |
|  | unknown | [885960..886205](+) | hypothetical protein |
|  | *bhlA* | [886240..886455](+) | hypothetical protein |
|  | *xhlB* | [886467..886733](+) | phage holin |
|  | unknown | [886789..887946](+) | N-acetylmuramoyl-L-alanine amidas |
|  | unknown | [887992..888939](-) | hypothetical protein |
|  | unknown | [889003..889557](-) | hypothetical protein |
|  | *ORF5* | [889847..890945](+) | tetratricopeptide repeat protein |
| G5 | unknown | [2159722..2159940](-) | hypothetical protein |
|  | *yopY* | [2160070..2160327](-) | hypothetical protein |
|  | *yosE* | [2160636..2160983](-) | hypothetical protein |
|  | *yosD* | [2160998..2161405](-) | hypothetical protein |
|  | unknown | [2162058..2162264](-) | hypothetical protein |
|  | unknown | [2162559..2162822](-) | hypothetical protein |
|  | *yorV* | [2163593..2163811](-) | hypothetical protein |
|  | *mtbP* | [2163862..2165355](-) | DNA (cytosine-5-)-methyltransferase |
|  | unknown | [2165422..2166258](-) | site-specific DNA-methyltransferase |
|  | unknown | [2166383..2166730](-) | hypothetical protein |
|  | *yorS* | [2166792..2167310](-) | 5'-3'-deoxyribonucleotidase |
|  | *yorR* | [2167288..2167815](-) | AAA family ATPase |
|  | *yorP* | [2167975..2168178](-) | YorP family protein |
|  | *yabE* | [2168190..2168897](-) | hypothetical protein |
|  | *yorL* | [2168924..2172853](-) | DNA polymerase III subunit alpha |
|  | *yorK* | [2172866..2174596](-) | single-stranded-DNA-specific exonuclease RecJ |
|  | *yorK* | [2174596..2175732](-) | hypothetical protein |
|  | *yorI* | [2175748..2177262](-) | hypothetical protein |
|  | *yorH* | [2177277..2177747](-) | hypothetical protein |
|  | *yorG* | [2177788..2178759](-) | ATP-binding protein |
|  | *yorF* | [2178849..2179763] | hypothetical protein |
|  | unknown | [2179786..2180166](-) | hypothetical protein |
|  | unknown | [2180305..2180688](-) | hypothetical protein |
|  | *yorC* | [2180880..2181257](-) | hypothetical protein |
|  | *yorA* | [2181296..2183038](-) | right-handed parallel beta-helix repeat-containing protein |
|  | *yoqZ* | [2183035..2183856](-) | hypothetical protein |
|  | unknown | [2184254..2184526](-) | hypothetical protein |
|  | unknown | [2184516..2185403](-) | hypothetical protein |
|  | unknown | [2185452..2185931](-) | hypothetical protein |
|  | *yopY* | [2185947..2186198](-) | hypothetical protein |
|  | *yoqX* | [2186224..2186469](-) | hypothetical protein |
|  | *yoqW* | [2186540..2187214](+) | SOS response-associated peptidase |
|  | *ligB* | [2187284..2188096](+) | ATP-dependent DNA ligase |
|  | unknown | [2188314..2188511](+) | hypothetical protein |
|  | unknown | [2188810..2189031](+) | hypothetical protein |
|  | unknown | [2189426..2189788](-) | hypothetical protein |
| G6 | *pgsE* | [3506842..3507003](-) | hypothetical protein |
|  | *pgsA* | [3507011..3508159](-) | CapA family protein |
|  | *capC* | [3508178..3508627](-) | poly-gamma-glutamate biosynthesis protein PgsC |
|  | *ywsC* | [3508642..3509823](-) | poly-gamma-glutamate synthase PgsB |

**Table S3 Endogenous promoters selected according to FPKM values**

| **weak promoter** | | **moderate promoter** | | **strong promoter** | |
| --- | --- | --- | --- | --- | --- |
| promoter^a^ | FPKM^b^ | promoter | FPKM | promoter | FPKM |
| PR*_ugt_* | 691 | PR*_gltX_* | 1268 | PR*_pgmi_* | 4143 |
| PR*_suc_* | 701 | PR*_nad_* | 1333 | PR*_hom_* | 4737 |
| PR*_ydh_* | 810 | PR*_arg_* | 1480 | PR*_hem_* | 7060 |
| PR*_accD_* | 890 | PR*_gltA_* | 1592 | PR*_ldh_* | 7569 |
| PR*_clp_* | 1024 | PR*_ahp_* | 2079 | PR*_rpsU_* | 16570 |
| PR*_tpxi_* | 1243 | PR*_nrfA_* | 2890 | PR*_alsD_* | 28940 |

^a^ The promoters were named according to the corresponding genes; for example, PR*_ugt_* represents the original promoter and RBS (ribosome binding site) of *ugt* gene.

^b^ FPKM value represents the transcriptional activity of a specific promoter.

**Table S4** **Plasmids used in this study**

| **Plasmids** | **Relative characteristics** | **source** |
| --- | --- | --- |
| pKSU | pKSV7 derivative with *upp* gene, temperature-sensitive replication origin, Ap^r^ (gram-negative) and Cm^r^ (gram-positive) | [24] |
| pKSU-ΔG1 | pKSU derivative, carrying deletion fragment of G1 | This work |
| pKSU-ΔG2 | pKSU derivative, carrying deletion fragment of G2 | This work |
| pKSU-ΔG3 | pKSU derivative, carrying deletion fragment of G3 | This work |
| pKSU-ΔG4 | pKSU derivative, carrying deletion fragment of G4 | This work |
| pKSU-ΔG5 | pKSU derivative, carrying deletion fragment of G5 | This work |
| pKSU-ΔG6 | pKSU derivative, carrying deletion fragment of G6 | This work |
| pKSU-Δ*itu* | pKSU derivative, carrying deletion fragment of *itu* | This work |
| pKSU-Δ*fenD* | pKSU derivative, carrying deletion fragment of *fenD* | This work |
| pHT01 | *E. coli*-*Bacillus* shuttle vector, Ap^r^ (gram-negative) and Cm^r^ (gram-positive) | This lab |
| pHT-P_43_-*gfp* | pHT01 derivative, used for providing *gfp* gene | This lab |
| pBBR1MCS-2 | expression plasmid used for providing *lac* promoter | This lab |
| pHT-PR*_lac_*-*gfp* | pHT01 derivative, containing PR*_lac_* promoter and *gfp* gene | This work |
| pHT-PR*_ugt_*-*gfp* | pHT01 derivative, containing PR*_ugt_* promoter and *gfp* gene | This work |
| pHT-PR*_suc_*-*gfp* | pHT01 derivative, containing PR*_suc_* promoter and *gfp* gene | This work |
| pHT-PR*_ydh_*-*gfp* | pHT01 derivative, containing PR*_ydh_* promoter and *gfp* gene | This work |
| pHT-PR*_accD_*-*gfp* | pHT01 derivative, containing PR*_accD_* promoter and *gfp* gene | This work |
| pHT-PR*_clp_*-*gfp* | pHT01 derivative, containing PR*_clp_* promoter and *gfp* gene | This work |
| pHT-PR*_tpxi_*-*gfp* | pHT01 derivative, containing PR*_tpxi_* promoter and *gfp* gene | This work |
| pHT-PR*_gltX_*-*gfp* | pHT01 derivative, containing PR*_gltX_* promoter and *gfp* gene | This work |
| pHT-PR*_nad_*-*gfp* | pHT01 derivative, containing PR*_nad_* promoter and *gfp* gene | This work |
| pHT-PR*_arg_*-*gfp* | pHT01 derivative, containing PR*_arg_* promoter and *gfp* gene | This work |
| pHT-PR*_gltA_*-*gfp* | pHT01 derivative, containing PR*_gltA_* promoter and *gfp* gene | This work |
| pHT-PR*_ahp_*-*gfp* | pHT01 derivative, containing PR*_ahp_* promoter and *gfp* gene | This work |
| pHT-PR*_nrfA_*-*gfp* | pHT01 derivative, containing PR*_nrfA_* promoter and *gfp* gene | This work |
| pHT-PR*_pgmi_*-*gfp* | pHT01 derivative, containing PR*_pgmi_* promoter and *gfp* gene | This work |
| pHT-PR*_hom_*-*gfp* | pHT01 derivative, containing PR*_hom_* promoter and *gfp* gene | This work |
| pHT-PR*_hem_*-*gfp* | pHT01 derivative, containing PR*_hem_* promoter and *gfp* gene | This work |
| pHT-PR*_ldh_*-*gfp* | pHT01 derivative, containing PR*_ldh_* promoter and *gfp* gene | This work |
| pHT-PR*_rpsU_*-*gfp* | pHT01 derivative, containing PR*_rpsU_* promoter and *gfp* gene | This work |
| pHT-PR*_alsD_*-*gfp* | pHT01 derivative, containing PR*_alsD_* promoter and *gfp* gene | This work |
| pKSU-PR*_suc_* | pKSU derivative, containing PR*_suc_* flanked by upstream and downstream regions of *srf* | This work |
| pKSU-PR*_tpxi_* | pKSU derivative, containing PR*_tpxi_* flanked by upstream and downstream regions of *srf* | This work |

Cm^r^, chloramphenicol resistance; Ap^r^, ampicillin resistance

**Table S5** **Primers used in this study**

| **Primer** | **Sequence (5’ to 3’)** |
| --- | --- |
| For construction of genome reduction | |
| G1-UF | CAAAATAAGTCGACTCTAGA**GGATCC**CCGTTCTAAAAATGATCTGAC |
| G1-UR | CCTTCCGCTCATTCAGTAATATACAGCCTCATCCT |
| G1-DF | GAGGCTGTATATTACTGAATGAGCGGAAGGTTTAT |
| G1-DR | TACGAATTCGAGCTC**GGTACC**TCATGTCTATGGCCTGCTGT |
| G2-UF | CAAAATAAGTCGACTCTAGA**GGATCC**TCGACGGAAATTGGCTGAACTGG |
| G2-UR | GGAAAGTTCCCGATGCCCATCCGGTTTAAATTCCTCTCC |
| G2-DF | CCCGAGCTCTTGGTCATACCCTTTACTGCAT |
| G2-DR | TACGAATTCGAGCTC**GGTACC**GCTTCAGTATCGGCTGTAACAATGGG |
| G3-UF | CAAAATAAGTCGACTCTAGA**GGATCC**GACCGTGCCAGAAATTCTGCGATAG |
| G3-UR | TGTCGTCTCTTCACCCGAAAGAGTGTCCCGGATATAC |
| G3-DF | CGGGACACTCTTTCGGGTGAAGAGACGACAGGCATGTACC |
| G3-DR | TACGAATTCGAGCTC**GGTACC**CATCATGGCGATGATCTCCTGATCC |
| G4-UF | CAAAATAAGTCGACTCTAGA**GGATCC**GGATCTCAATAAAGTCTGTCCCGGGTAAAG |
| G4-UR | CTGCTGCAGAAGTCCAGATCCACTTCGACGGCTTTG |
| G4-DF | CTGCTGCAGAAGTCCGGACTTCTGCAGCAGGATCAGAG |
| G4-DR | TACGAATTCGAGCTC**GGTACC**GAAACCCTATTATATCAGTGTTCCG |
| G5-UF | CAAAATAAGTCGACTCTAGA**GGATCC**GATCGTTCATCCTTCCCGGTTCCTCGAA |
| G5-UR | ATATGGAGGAAAGTCGAACGAAGCAGATAAGCCGAAGGTG |
| G5-DF | TTATCTGCTTCGTTCGACTTTCCTCCATATCACCTATGGC |
| G5-DR | TACGAATTCGAGCTC**GGTACC**GACATTTGTGAAGGGCACGCAT |
| G6-UF | CAAAATAAGTCGACTCTAGA**GGATCC**AAAAAATATGCCGGCAAGCGGAAGC |
| G6-UR | GCCGCAGAGCGGAATAGCTAAACGGAATGCGCCGGGTG |
| G6-DF | GCATTCCGTTTAGCTATTCCGCTCTGCGGCTTTTTCTTT |
| G6-DR | TACGAATTCGAGCTC**GGTACC**GGTTACTCATTATAGCCTGTGCTG |
| For genetic modification of GR167 | |
| *itu-*UF | TTTGGAACAAAATAA**GGATCC**AAATTGAGGCAATAGGAATAG |
| *itu*-UR | TAACAGTCAGTGTGTTGGGATCGTTTGCGGGAGAC |
| *itu*-DF | GCAAACGATCCCAACACACTGACTGTTAAAATAGC |
| *itu*-DR | CTTGCATGCCTGCAG**GTCGAC**TGGGGGCTTCACAATGATTTATGT |
| *itu*-JF | GCGATTGATGGTGCCCATGAC |
| *itu*-JR | AAGTTCCGCGTCATGATTCC |
| *fenD*-UF | TTTGGAACAAAATAA**GGATCC**CTATCTTGCCCTCTGTCTTC |
| *fenD-*UR | AGAAATATCCTTACGCAAACGGCAAAGTGGACC |
| *fenD*-DF | TTTGCCGTTTGCGTAAGGATATTTCTGGTGCCG |
| *fenD*-DR | CTTGCATGCCTGCAG**GTCGAC**TTGAAGAATACTGTTTATGCTT |
| *fenD*-JF | AATGGGTCAGCCGGTAGCTGGCAAG |
| *fenD*-JR | TGCGTCAAATTCAGGGGAAACATCG |
| *srf*-UF | CAAAATAAGTCGACTCTAGA**GGATCC**CGCACAAAATTCTTGTGAATCAGC |
| *srf*-UR | TCCCTTTTTAGATCTAAGATAAAATTGTCAATTCTTCCTTAGAAACAG |
| *srf-*DF | TTTTATCTTAGATCTAAAAAGGGAGGCGTACACATATGGG |
| *srf*-DR | CTTGCATGCCTGCAG**GTCGAC**GAACCGCTTCGACTGCACATATTCC |
| *suc-*F | TGACAATTTTATCTTAAGAGCAGACTCGGTTAAGAAG |
| *suc-*R | TACGCCTCCCTTTTTTTCCCATCCTCCTAACTTATGT |
| *tpxi-*F | TGACAATTTTATCTTGAGAAAAGTTAACTGCCGCCC |
| *tpxi-*R | TACGCCTCCCTTTTTTCTGATTCCTCCCTTATCTATGT |
| Promoter-JF | GGATTTCGGCGGTGTTTTGAATCGG |
| Promoter-JR | GCGGATAGAATTTCTCTGTATACC |
| *srf*-p-JF | AGGAATTGACGCTTGCCGTGATAGG |
| *srf*-p-JR | GAATCCGGCCGTACAGGTCATGAGG |
| For construction of endogenous promoter library | |
| *gfp*-F | CACCGGAATTAGCTT**GGTACC**ATGAGTAAAGGAGAAGAACT |
| *gfp*-R | GACGTCGAC**TCTAGA**TTTGTATAGTTCATCCATGC |
| PR*_lac_*-*gfp-*F | CACCGGAATTAGCTT**GGTACC**TTTACACTTTATGCTTCCGG |
| PR*_lac_*-*gfp*-R | TTCTCCTTTACTCATCAACATACGAGCCGGAAGCA |
| PR*_ugt_*-*gfp-*F | CACCGGAATTAGCTT**GGTACC**TTTTTGTTTTGAGTGATACGGT |
| PR*_ugt_*-*gfp*-R | TTCTCCTTTACTCATGTAAATTCACCTCAATGTAA |
| PR*_suc_*-*gfp-*F | CACCGGAATTAGCTT**GGTACC**AAGAGCAGACTCGGTTAAGAAG |
| PR*_suc_*-*gfp*-R | TTCTCCTTTACTCATTTCCCATCCTCCTAACTTATGT |
| PR*_ydh_*-*gfp-*F | CACCGGAATTAGCTT**GGTACC**ACTCTCTGTATAAAAGCCAT |
| PR*_ydh_*-*gfp*-R | TTCTCCTTTACTCATAATCTTCTCCTCCATTTAGA |
| PR*_accD_*-*gfp-*F | CACCGGAATTAGCTT**GGTACC**TAACACAAAAATGAAATGGCAGCGCG |
| PR*_accD_*-*gfp*-R | TTCTCCTTTACTCATATGATTACCTCCCTTTTGTGAAGG |
| PR*_clp_*-*gfp-*F | CACCGGAATTAGCTT**GGTACC**TCAGGTGCGGTGCTGACG |
| PR*_clp_*-*gfp*-R | TTCTCCTTTACTCATAATGCTCCTCCTTCACC |
| PR*_tpxi_*-*gfp-*F | CACCGGAATTAGCTT**GGTACC**GAGAAAAGTTAACTGCCG |
| PR*_tpxi_*-*gfp*-R | TTCTCCTTTACTCATTCTGATTCCTCCCTTATC |
| PR*_gltX_*-*gfp-*F | CACCGGAATTAGCTT**GGTACC**AATCGGACAAGGATTTGATGTG |
| PR*_gltX_*-*gfp*-R | TTCTCCTTTACTCATGTTCAAATACTTCCTTTCATCTCGTC |
| PR*_nad_*-*gfp-*F | CACCGGAATTAGCTT**GGTACC**ACAGGCCGTCTCCTATCCGTTTC |
| PR*_nad_*-*gfp*-R | TTCTCCTTTACTCATACATCACCCTCCTGTTTTATTTACACC |
| PR*_arg_*-*gfp-*F | CACCGGAATTAGCTT**GGTACC**ACGTATAAAAAGGCTCTGCTCCGC |
| PR*_arg_*-*gfp*-R | TTCTCCTTTACTCATGTTAAAAGCTCCCTTTG |
| PR*_gltA_*-*gfp-*F | CACCGGAATTAGCTT**GGTACC**TGCGTCCACACCCATCTATC |
| PR*_gltA_*-*gfp*-R | TTCTCCTTTACTCATAATTCCTCTCCCCCGATCAG |
| PR*_ahp_*-*gfp-*F | CACCGGAATTAGCTT**GGTACC**GACCATACCCCCTTTGGTTTATAC |
| PR*_ahp_*-*gfp*-R | TTCTCCTTTACTCATAATATCTTCCTCCTGAAATGTG |
| PR*_nrfA_*-*gfp-*F | CACCGGAATTAGCTT**GGTACC**TGTCAGACGGCTCGTTTTTCG |
| PR*_nrfA_*-*gfp*-R | TTCTCCTTTACTCATATAAAAGCCTCCTTCTCTATATATCATATCCG |
| PR*_pgmi_*-*gfp-*F | CACCGGAATTAGCTT**GGTACC**GACTCAAACGTTCCTTCCTATTC |
| PR*_pgmi_*-*gfp*-R | TTCTCCTTTACTCATAAGCGTTTTACGACAAAATCCCG |
| PR*_hom_*-*gfp-*F | CACCGGAATTAGCTT**GGTACC**AGAAACTCCACCTTTCTCCCTTTTG |
| PR*_hom_*-*gfp*-R | TTCTCCTTTACTCATGAAGATATGACGTAAAAATATTTGG |
| PR*_hem_*-*gfp-*F | CACCGGAATTAGCTT**GGTACC**CCTGTCACCTACCCGCAGTAAAG |
| PR*_hem_*-*gfp*-R | TTCTCCTTTACTCATAGCCGTTCATGCACCCCCGTACATC |
| PR*_ldh_*-*gfp-*F | CACCGGAATTAGCTT**GGTACC**GATTCATTATTCTCCTTTCGTAC |
| PR*_ldh_*-*gfp*-R | TTCTCCTTTACTCATGTATGCTCATCCTCCAGTGTTTC |
| PR*_rpsU_*-*gfp-*F | CACCGGAATTAGCTT**GGTACC**ACGAAACACCCCTTTCCGTCAT |
| PR*_rpsU_*-*gfp*-R | TTCTCCTTTACTCATTCTCTTTCCCTCCCTCCGAATAC |
| PR*_alsD_*-*gfp-*F | CACCGGAATTAGCTT**GGTACC**CCTTATCCATTCCTTTCCCTTTG |
| PR*_alsD_*-*gfp*-R | TTCTCCTTTACTCATCACCCTCACTCCTTATTATGCATAT |
| For RT-qPCR | |
| Q*gfp*-F | TGCACTACTGGAAAACTACC |
| Q*gfp*-R | ACTCGATGCGATTAACAAGG |
| Q*rpsU*-F | GTCGTTAGAAAAAACGAATCGCTTG |
| Q*rpsU*-R | TTGCGTTTTCTAGCAGCTTCTGACT |
| Q*srf*-F | TTTCAAACCTTGCCGGCTTC |
| Q*srf*-R | ACAATCGCAGCCGGATTAAG |

The restriction sites are indicated in bold.

**Sequence of the promoters of PR*_suc_* and PR*_tpxi_***

**PR*_suc_*:**

AAGAGCAGACTCGGTTAAGAAGAGCTGTCGCCCTGCATTACGACCGGCAGAAAGATCAAGCCCCGAAGGTTATCGCAACAGGCAGGGGGCATGTGGCGGAAAATATCATAAAAGAAGCTGAGAAGGCGGGGGTCCCGATTCAGGAAGACAGGACCCTTGTCGAATTAATGCGGCATTTGACGGTCGACGATCAGATACCGGAAGCGCTTTATGAAACCGTCGCTGAAATTTTTTCATTTGTTTACCGGCTGGATGAAAGCCTGAAAAACGAAAAATAAGGATCAATCATTTCCCAGACTCCGTTTAAATTTATATTTTCAATAAAATAAAAGTTAGAATGTTTGGAAGGATATAAAGATTTTGTTTTGAACCCTAGACAATTCTTCCAGTATTATATAGAATGAAAGCGC

AGTCTATTTTTAGTTTTGCTACATAAGTTAGGAGGATGGGAA

| **Start** | **End** | **Score** | **Predicted promoter sequences** |
| --- | --- | --- | --- |
| 298 | 343 | 0.98 | CTCCGTTTAAATTTATATTTTCAATAAAATAAAAGTTAGAATGTTTGGAA |
| 328 | 373 | 0.87 | AAAAGTTAGAATGTTTGGAAGGATATAAAGATTTTGTTTTGAACCCTAGA |
| 361 | 406 | 0.99 | TTGTTTTGAACCCTAGACAATTCTTCCAGTATTATATAGAATGAAAGCGC |

**PR*_tpxi_*:**

GAGAAAAGTTAACTGCCGCCCGCAGTTAGCTTTTTTTACGATGCGGCATAACCTTTGCAGTTTGCCTGAAACTCGACTATGATAGAGCTATACATAGATAAGGGAGGAATCAGA

| **Start** | **End** | **Score** | **Predicted promoter sequences** |
| --- | --- | --- | --- |
| 49 | 94 | 0.95 | TAACCTTTGCAGTTTGCCTGAAACTCGACTATGATAGAGCTATACATAGA |
| 60 | 105 | 0.94 | GTTTGCCTGAAACTCGACTATGATAGAGCTATACATAGATAAGGGAGGAA |
